# Impact of elevated anti-apoptotic MCL-1 and BCL-2 on the development and treatment of *MLL-AF9* AML in mice

**DOI:** 10.1038/s41418-018-0209-1

**Published:** 2018-11-23

**Authors:** Natasha S. Anstee, Rebecca A. Bilardi, Ashley P. Ng, Zhen Xu, Mikara Robati, Cassandra J. Vandenberg, Suzanne Cory

**Affiliations:** 1 Walter and Eliza Hall Institute of Medical Research, Melbourne, Victoria 3052 Australia; 2 Department of Medical Biology, University of Melbourne, Melbourne, Victoria 3010 Australia; 3 Present Address: Deutsches Krebsforschungszentrum (DKFZ), Experimental Hematology Division, 69120 Heidelberg, Germany

**Keywords:** Experimental models of disease, Cancer models

## Abstract

Many acute myeloid leukaemias (AMLs) express high levels of BCL-2 and MCL-1, especially after therapy. To test the impact of these anti-apoptotic proteins on AML development and treatment, we used haemopoietic reconstitution to generate *MLL-AF9* AMLs expressing *BCL-2* or *Mcl-1* transgenes. AMLs with elevated BCL-2 or MCL-1 had a higher proportion of mature myeloid cells but, like conventional *MLL-AF9* AMLs, were readily transplantable. Short-term cell lines established from multiple primary AMLs of each genotype were tested in vitro for susceptibility to chemotherapeutics currently used for treating AML (daunorubicin, etoposide, cytarabine); the proteasome inhibitor bortezomib; CDK7/9 inhibitors; and BH3 mimetics, which bind and inhibit pro-survival proteins. The BH3 mimetics tested, alone and in combination with the other drugs, were: ABT*-*737 which, like its clinical counterpart navitoclax, targets BCL-2, BCL-X_L_ and BCL-W; BCL-2-specific ABT-199 (venetoclax); BCL*-*X_L_*-*specific A-1331852; and S63845, a new MCL-1-specific BH3 mimetic. As single agents, daunorubicin and bortezomib had the greatest efficacy. Elevated MCL-1 or BCL-2 reduced sensitivity to daunorubicin but, surprisingly, not to bortezomib. MCL-1 markedly enhanced resistance to ABT-737 and ABT-199 but not S63845, and BCL-2 increased resistance to S63845 but not to ABT-737 or ABT-199. Notable synergies were achieved by combining BH3 mimetics with daunorubicin: S63845 increased the sensitivity of both MCL-1 and BCL-2 overexpressing *MLL-AF9* AMLs, and ABT-737 aided in killing those overexpressing BCL-2. Synergy between daunorubicin and ABT-199 was also apparent in vivo, although not curative. Impressive synergistic responses were achieved for human *MLL*-fusion AML cell lines treated with daunorubicin plus either ABT-737, ABT-199 or S63845, and with ABT-199 plus S63845, with or without daunorubicin. Our data suggest that AML patients may benefit from combining conventional cytotoxic drugs with BH3 mimetics targeting BCL-2 or MCL-1 or, if tolerated, both these agents.

## Introduction

Acute myeloid leukaemia (AML) is a relentless proliferation of myeloid cells that results in catastrophic bone marrow failure. Although AML can arise from diverse types of genetic changes, individual tumours generally carry only two to four driver mutations [1]. Balanced chromosomal translocation/inversion events giving rise to chimaeric fusion genes are common in AML, particularly in children and younger adults and in therapy-induced AML. Many of these involve the *MLL* (mixed lineage leukaemia) gene located on chromosome 11 band q23, which encodes a large multi*-*domain epigenetic regulator [2]. The translocation breakpoint separates the N*-*terminal DNA binding domain of MLL from its C-terminal histone H3 lysine 4 (H3K4) methyl transferase domain, thereby disabling its methyl transferase activity. The eight genes that most frequently recombine with *MLL* in translocations encode proteins involved in multi-component transcription elongation complexes [3]. Therefore, most MLL translocations, including the t(9;11) that produces the *MLL-AF9* fusion gene, deregulate transcription of MLL target genes [4, 5].

Many AML patients have a dismal prognosis and more effective therapies are sorely needed [6]. Standard treatment involves administration of cytarabine (ara-C) together with an anthracycline (usually daunorubicin or idarubicin) and sometimes also etoposide [6]. While cytarabine interferes with DNA replication, provoking premature chain termination [7], the anthracylines and etoposide inhibit topoisomerase II, increasing the frequency of double-stranded breaks [8]. Anthracyclines are also believed to generate reactive oxygen species and inhibit DNA and RNA synthesis [9]. All these agents invoke apoptosis via the intrinsic (also called mitochondrial) apoptosis pathway, which is regulated by the BCL-2 protein family.

BCL-2 family members serve as a cellular life/death switch (reviewed in ref. [10]). BCL-2 and its closest relatives (BCL-X_L_, BCL-W, MCL-1 and A1/BFL-1) promote cell survival by preventing activation of structurally similar but pro*-*apoptotic relatives BAX and BAK on the mitochondrial outer membrane (MOM). Stress signals, such as DNA damage or oncogene expression, upregulate distant relatives known as BH3*-*only (BCL-2 homology domain 3-only) proteins, which bind tightly via their BH3 domains to the hydrophobic surface groove of the pro-survival proteins, thereby preventing their restraint of BAX and BAK. Certain BH3-only proteins (BIM, tBID, PUMA and possibly others) can also bind transiently to the surface groove of BAX and BAK, inducing a major conformational change that prompts homo*-*dimerisation. BAX/BAK homo-dimers aggregate to form homo-oligomeric pores, through which cytochrome c egresses and initiates activation of caspases that cleave vital cellular proteins.

Many AMLs express high levels of pro-survival BCL-2 or MCL-1 [11–13], especially upon relapse after therapy [14]. Importantly, using conditional gene deletion in mice, MCL-1 has been shown to be essential for the development and sustained growth of AML induced by *c-Myc* or *MLL*-fusion genes [11, 15]. These observations led us to test the impact of overexpression of MCL-1 or BCL-2 on the development and treatment of *MLL-AF9* AML in mice. In particular, we were keen to test responsiveness to recently developed BH3 mimetics (drugs that mimic BH3-only proteins) and to agents reported to downregulate MCL-1, such as CDK7/9 inhibitors [16, 17] and the proteasome inhibitor bortezomib [18], which is being trialled clinically for AML [19].

## Results

### Generation of murine *MLL-AF9* AMLs overexpressing BCL-2 or MCL-1

**Fig. 1:**
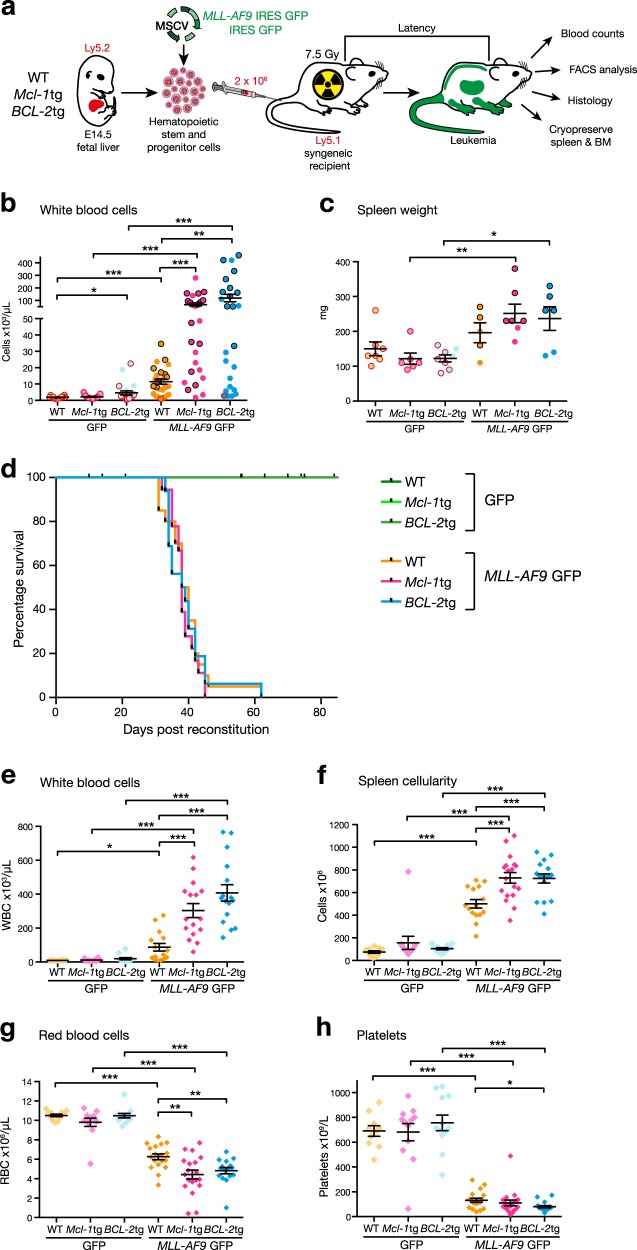
Impact of overexpression of MCL-1 or BCL-2 on the development of *MLL-AF9* AML. **a** Generation of *MLL-AF9* AMLs. Haemopoietic stem and progenitor cells from foetal livers of E14.5 WT, vavP-*Mcl-1*tg or vavP-*BCL-2*tg embryos were infected with either *MLL-AF9*/GFP or control GFP MSCV virus and transplanted into sublethally irradiated (7.5 Gy) Ly5.1 recipients (2 × 10^6^ cells/mouse). Mice developing leukaemia were autopsied, subjected to haemopoietic and histologic analysis and their spleen and bone marrow cells were cryopreserved. **b, c** Overexpression of MCL-1 or BCL-2 provokes early elevation of myeloid cells. 3 wk analysis of (**b**) blood leucocytes and (**c**) spleen weight in mice reconstituted with WT/GFP (light orange, *n* = 7–18), *Mcl-1*tg/GFP (light pink, *n* = 6–19), *BCL-2*tg/GFP (light blue, *n* = 8–22), WT/*MLL-AF9* (orange, *n* = 5–27), *Mcl-1*tg/*MLL-AF9* (pink, *n* = 7*–*26) or *BCL-2*tg/*MLL-AF9* (blue, *n* = 6–25) cells. Each dot represents one mouse and mean ± S.E.M is indicated. *p < 0.05, **p < 0.01, ***p < 0.001, calculated by Student’s *T-*test with Welch’s correction. **d** Overexpression of MCL-1 or BCL-2 does not affect morbidity. Kaplan–Meier plot showing the survival of mice transplanted with WT/*MLL-AF9* (orange, *n* = 20), *Mcl-1*tg/*MLL-AF9* (pink, *n* = 18), *BCL-2*tg/*MLL-AF9* (blue, *n* = 17) or GFP control (green, *n* = 12–13) cells. Mice were monitored regularly and euthanased when their symptoms dictated ethical endpoint. Control GFP mice remained healthy until culled (70–90 d). **e**–**h** Overexpression of MCL-1 or BCL-2 exacerbates the *MLL-AF9* phenotype. Enumeration of (**e**) blood leucocytes, (**f**) spleen cells, (**g**) red blood cells and (**h**) platelets in sick mice reconstituted with either WT/GFP (light orange, *n* = 10–12), *Mcl-1*tg/GFP (light pink, *n* = 12), *BCL-2*tg/GFP (light blue, *n* = 12), WT/*MLL-AF9* (orange, *n* = 15–18), *Mcl-1*tg/*MLL-AF9* (pink, *n* = 16–19) or *BCL-2*tg/*MLL-AF9* (blue, *n* = 16) cells. Each dot represents one mouse; mean ± S.E.M is indicated. *p < 0.05, **p < 0.01, ***p < 0.001, calculated by Student’s *T*-test with Welch’s correction

We have previously developed transgenic mice with pan*-*haemopoietic overexpression of human BCL-2 or mouse MCL-1 protein [20, 21]. To generate *MLL-AF9* AMLs, foetal liver haemopoietic stem and progenitor cells (HSPCs) from these and WT mice (all C57BL/6-Ly5.2) were infected with *MLL-AF9*/*GFP* or *GFP* (control) retroviruses and transplanted into sublethally irradiated C57BL/6-Ly5.1 recipient mice (Fig. 1a). For brevity, the reconstituted mice are designated hereafter according to the genotype of the donor foetal liver cells and the virus used (e.g. WT/*GFP* indicates mice reconstituted with WT foetal liver cells infected with control *GFP* virus and *Mcl-1*tg*/MLL-AF9* indicates mice reconstituted with *Mcl-1*tg foetal liver cells infected with *MLL-AF9/GFP* virus).

### Overexpression of MCL-1 or BCL-2 exacerbates *MLL-AF9* AML

Three weeks after reconstitution, most *MLL-AF9* mice, especially those transplanted with infected cells from *Mcl-1*tg or *BCL-2*tg mice, had elevated white blood cell counts and enlarged spleens compared to the corresponding control *GFP* mice (Fig. 1b, c). Even at this early time point, the blood, spleen and bone marrow of the *MLL-AF9* mice was replete with donor-derived provirus-expressing (Ly5.2^+^GFP^+^) cells having a myeloid (Mac1^+^) phenotype (Supplementary Figure S1 and Table S1).

Despite provoking more severe early leukocytosis, overexpression of MCL-1 or BCL-2 did not accelerate morbidity (Fig. 1d). Irrespective of whether their reconstituting stem/progenitor cells were WT, *Mcl-1*tg or *BCL-2*tg in genotype, all *MLL-AF9* mice required ethical euthanasia within 60 days, whereas the corresponding control *GFP* mice remained healthy until they were culled (70–90 d). The spleen, bone marrow and blood of the sick mice were dominated by donor-derived (Ly5.2^+^GFP^+^) myeloid (Mac1^+^Gr1^-^ and Mac1^+^Gr1^+^) cells (Supplementary Figure S2). Of note, the AML phenotype appeared more extreme in terminally ill *Mcl-1*tg*/MLL-AF9* and *BCL-2*tg*/MLL-AF9* mice than in WT/*MLL-AF9* mice; leukocytosis and splenomegaly were more severe (Fig. 1e, f) and anaemia and thrombocytopenia more pronounced (Fig. 1g, h). Histological analysis (Supplementary Figure S3) revealed total effacement of the bone marrow, disruption of splenic architecture and leucocyte infiltration of organs such as kidney, pancreas and liver.

**Fig. 2:**
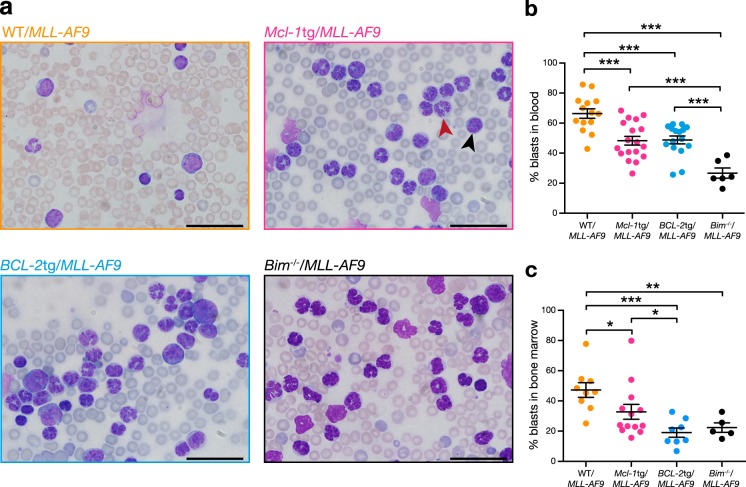
Leukocytosis in sick primary AML mice. **a** Blood smears typical of mice reconstituted with stem/progenitor cells of the following genotypes: WT/*MLL-AF9* (mouse #2036), *Mcl-1*tg/*MLL-AF9* (mouse #2039), *BCL-2*tg/*MLL-AF9* (mouse #2041) and *Bim*^-/-^/*MLL-AF9* (mouse #1250). Scale Bars: 20 μm, ×1000 magnification. A typical blast cell is indicated by the black arrowhead and a differentiated myeloid cell with a red arrowhead. (See Supplementary Figure S4 for bone marrow cytospins). **b, c** Quantification of blast cells (indicated by high nucleus to cytoplasm ratio, scored blinded) in (**b**) blood and (**c**) bone marrow of sick primary WT/*MLL-AF9* (orange; blood *n* = 14, BM *n* = 9), *Mcl-1*tg/*MLL-AF9* (pink; blood *n* = 18, BM *n* = 13), *BCL-2*tg/*MLL-AF9* (blue; blood *n* = 16, BM *n* = 8) and *Bim*^*-/-*^*/MLL-AF9* (black; blood *n* = 6, BM *n* = 5) mice. Each dot represents one mouse *p < 0.05, **p < 0.01, ***p < 0.001 calculated by Student’s *T-*test with Welch’s correction

In general, sick *Mcl-1*tg*/MLL-AF9* and *BCL-2*tg*/MLL-AF9* mice had a higher proportion of mature myeloid cells and a lower proportion of blasts than sick WT/*MLL-AF9* mice, as evidenced by blood smears, bone marrow cytospins and flow cytometry (Fig. 2, Supplementary Figures S2, S4, S5a and Table S2). Primary *MLL-AF9* AMLs lacking expression of the BH3-only protein BIM [22] had a similar phenotype (Fig. 2b, Supplementary Figure S4). We infer that the more severe leukocytosis in *Mcl-1*tg*/MLL-AF9, BCL-2*tg*/MLL-AF9* and *Bim*^*−/−*^*/MLL-AF9* AML-bearing mice reflects enhanced survival of maturing myeloid cells.

Transplantability tests in non-irradiated congenic mice (Supplementary Table S3) confirmed that all three genotypes were fully malignant. Of note, the transplanted secondary *Mcl-1*tg*/MLL-AF9* and *BCL-2*tg*/MLL-AF9* AMLs still had a greater proportion of differentiated cells than transplanted WT/*MLL-AF9* AMLs but the tertiary transplants were all dominated by undifferentiated blasts (Supplementary Figure S5).

### Expression of BCL-2 protein family members in primary AMLs

**Fig. 3:**
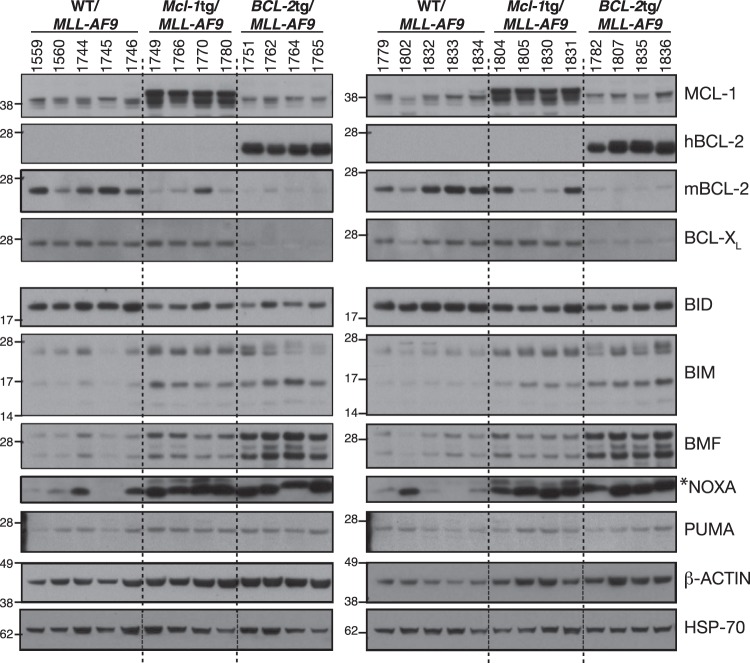
Expression of BCL-2 protein family members in primary *MLL-AF9* AMLs. Western blot analysis of bone marrow cells from sick primary mice. Lysates run in each lane derived from an individual mouse (mouse numbers shown). Blots are representative of four experiments. In the NOXA blot, NOXA is the more prominent lower band, as determined by probing lysates from *Noxa*^−*/*−^ control cells (not shown) and *indicates a non-specific second band. Molecular weight markers are indicated (kD)

Western blot analysis was performed on bone marrow cells from 8 to 10 AML-bearing mice of each genotype (Fig. 3). All WT/*MLL-AF9* AML samples had readily detectable levels of endogenous MCL-1, BCL-2 and BCL-X_L_ (A1 and BCL-W were not analysed). Endogenous MCL-1 levels were comparable between WT/*MLL-AF9* and *BCL-2*tg/*MLL-AF9* AMLs (size overlap with apparent degradation products of transgenic protein prevented the assessment of endogenous MCL-1 in *Mcl-1tg/MLL-AF9* AMLs). Interestingly, endogenous BCL-2 and BCL-X_L_ were lower in the *BCL-2*tg/*MLL-AF9* AMLs than in WT/*MLL-AF9* AMLs. By comparison, although 5 of 8 *Mcl-1tg/MLL-AF9* AMLs analysed had lower endogenous BCL-2 than the WT AMLs, none had lower BCL-X_L_.

Analysis for five BH3-only proteins (Fig. 3, lower panels) showed that all ten WT/*MLL-AF9* AMLs expressed relatively uniform levels of BID, PUMA, BIM and (with one exception) BMF, but NOXA expression was prominent in only four. Of note, BIM, BMF and NOXA levels were higher in *Mcl-1*tg*/MLL-AF9* and *BCL-2*tg*/MLL-AF9* than WT/*MLL-AF9* tumours.

### Impact of MCL-1 or BCL-2 on drug sensitivity

**Fig. 4:**
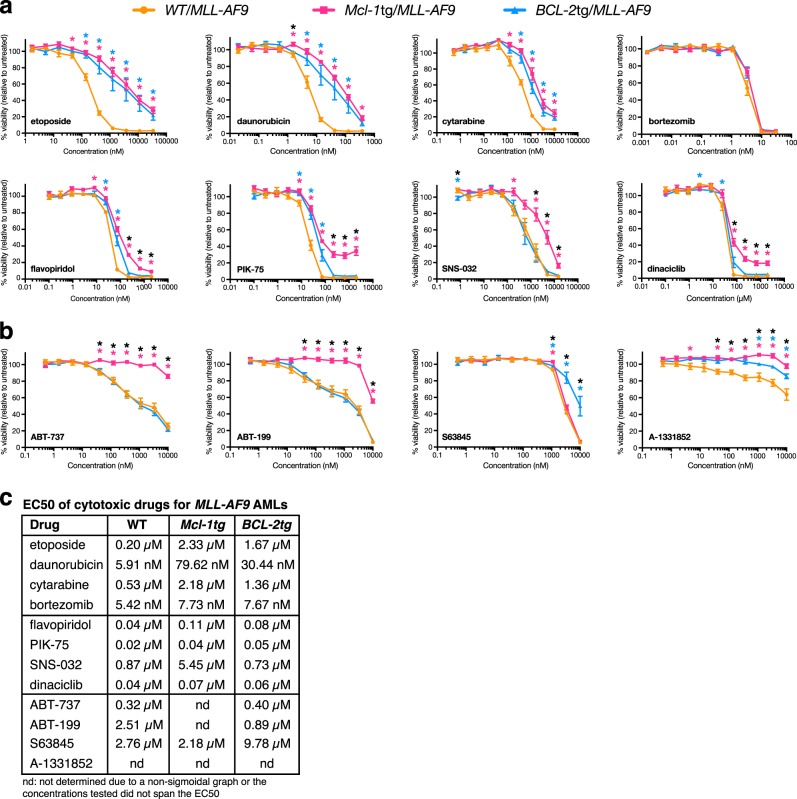
Drug sensitivity of cultured primary *MLL-AF9* AMLs. Dose-response of WT/*MLL-AF9* (orange), *Mcl-1*tg/*MLL-AF9* (pink) and *BCL-2*tg/*MLL-AF9* (blue) AMLs treated for 24 h with **a** chemotherapeutic drugs etoposide, daunorubicin, cytarabine, the proteasome inhibitor bortezomib, and CDK7/9 inhibitors flavopiridol, PIK-75, SNS-032 and dinaciclib and **b** BH3 mimetics ABT-737 (inhibits BCL-2, BCL-X_L_, BCL-W), ABT-199 (inhibits BCL-2), S63845 (inhibits MCL-1) and A*-*1331852 (inhibits BCL-X_L_). Cell viability was measured using CellTiter-Glo Luminescent Assay and indicated relative to untreated control cells (See also Supplementary Figure S6 for viability measured as % annexin V^–^PI^–^ double negative cells). 2–6 technical replicates per independent tumour were averaged and data plotted are average response of 5 independent AMLs per genotype ± S.E.M. *p < 0.05, calculated by Student’s *T-*test with Welch’s correction (pink asterisk indicates significant difference between WT/*MLL-AF9* versus *Mcl-1*tg/*MLL-AF9*; blue asterisk indicates significant difference between WT/*MLL-AF9* versus *BCL-2*tg/*MLL-AF9*; black asterisk indicates significant difference between *Mcl-1*tg/*MLL-AF9* versus *BCL-2*tg/*MLL-AF9*). Short-term cell lines were derived by culturing bone marrow cells from the following sick primary mice: WT/*MLL-AF9*: #1746, #1802, #1833, #1834, #2036; *Mcl-1*tg/*MLL-AF9*: #1750, #1770, #1780, #1805, #2038; *BCL-2*tg/*MLL-AF9*: #1751, #1763, #1765, #1807, #2040. **c** EC50s of cytotoxic drugs for primary *MLL-AF9 AMLs*. Values were calculated using GraphPad Prism by log-transforming the data and fitting it to a nonlinear regression

To gauge drug sensitivity, short-term cell lines were established from multiple (≥5) independent primary tumours of each genotype. In addition to standard genotoxic agents (cytarabine, daunorubicin and etoposide), we tested the proteasome inhibitor bortezomib, four CDK7/9 inhibitors, which inhibit transcription [23] and thereby diminish the level of short-lived proteins such as MCL*-*1 [17], and four BH3 mimetics having different specificities: ABT-737, which binds BCL-2, BCL-X_L_ and BCL-W; [24] BCL-2-specific ABT-199; [25] MCL*-*1*-*specific S63845; [26] and BCL-X_L_-specific A*-*1331852 [27]. Cell viability was determined at 24 h by measuring metabolic activity (CellTiter-Glo luminescence) (Fig. 4) and, for most agents, by enumerating cells excluding annexin V and propidium iodide (Supplementary Figure S6). Inhibition of cell death by the pan-caspase inhibitor Q-VD-OPh confirmed that the drugs acted by inducing apoptosis (Supplementary Figure S6c–e).

As single agents, daunorubicin was the most potent of the genotoxic drugs (Fig. 4c) and overexpression of either MCL-1 or BCL-2 increased resistance 13.5 and 5.1 fold respectively. Bortezomib was as active against the WT/*MLL-AF9* AMLs as daunorubicin but, perhaps surprisingly, overexpression of BCL-2 or MCL-1 did not confer resistance. CDK inhibitors flavopiridol, PIK-75 and dinaciclib were more potent than SNS-032 and, while overexpression of MCL-1 or BCL-2 increased resistance, this was more apparent in the apoptosis (Supplementary Figure S6) than the metabolic assay (Fig. 4).

**Fig. 5:**
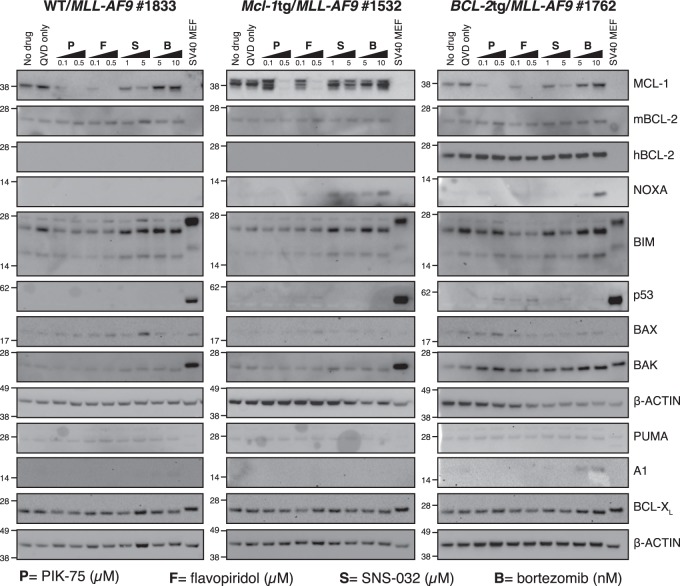
Impact of CDK inhibitors and bortezomib on apoptosis regulators in primary AML cell lines. Western blot analysis of expression of BCL-2 family proteins and p53 in cell lines derived from primary AMLs: WT/*MLL-AF9* (#1833 left panel), *Mcl-1*tg/*MLL-AF9* (#1532; central panel) and *BCL-2*tg/*MLL-AF9* (#1762; right panel). Cells were incubated for 6 h with no drug, Q-VD-OPh (QVD) only or QVD plus PIK-75 (P), flavopiridol (F), SNS-032 (S) or bortezomib (B) at doses indicated (in μM for CDK inhibitors and nM for bortezomib). Representative blots of two experiments with independent tumours (see Supplementary Figure S7 for western blots of WT/MLL-AF9 #1559, *Mcl-1*tg/*MLL-AF9* #1766 and *BCL-2*tg/*MLL-AF9* #1806). SV40 transformed mouse embryo fibroblasts (MEFs) served as a positive control for p53 and β-ACTIN as a loading control. Molecular weight markers are indicated (kD)

To assess the impact of the CDK inhibitors and bortezomib on apoptosis regulators, western blot analysis was performed on two AML lines of each genotype following drug exposure for 6 h in the presence of Q-VD-OPh (Fig. 5, Supplementary Figure S7). PIK-75, flavopiridol and SNS-032 reduced MCL-1 levels in each genotype in a dose-dependent manner, even in the *Mcl-1*tg/*MLL-AF9* tumours. Responsiveness varied, but in general SNS-032 was less effective at reducing MCL-1 than PIK-75 and flavopiridol. SNS-032 treatment increased NOXA in one *Mcl-1*tg/*MLL-AF9* tumour but not the other.

Bortezomib treatment did not decrease MCL-1 in any of the *MLL-AF9* AMLs, in contrast to certain other cell types [18, 28]. Indeed, levels rose in several, despite the increased NOXA, which would be expected to bind and facilitate proteasome*-*mediated degradation [29]. Q-VD-OPh may have precluded caspase*-*mediated degradation of MCL-1 triggered by the proteasome inhibitor [30]. Alternatively, MCL-1 in AML cells may be bound to BIM, which prevents MCL-1 degradation rather than enhancing it [31].

p53 was low to undetectable in the three treated AMLs analysed, suggesting that it is probably wild type, as is common in human AMLs [32].

### Sensitivity of *MLL-AF9* AMLs to BH3 mimetics

As single agents, the BH3 mimetics were not highly active against the *MLL-AF9* AMLs, BCL-X_L_-specific A-1331852 being the least active (Fig. 4b, c and Supplementary Figure S6b). Importantly, however, *MLL-AF9* AMLs overexpressing BCL-2 (blue) were just as sensitive as WT-*MLL-AF9* AMLs (orange) to ABT-737 and ABT-199, while highly resistant to S63845. Conversely, although those overexpressing MCL-1 (pink) were very resistant to ABT-737 and ABT-199, as expected, they were just as sensitive to S63845 as WT*/MLL-AF9* AMLs, even though S63845 has a 6-fold lower affinity for mouse as compared to human MCL-1 [26].

To test whether the BH3 mimetics could synergise with any of the drugs under test, we treated 5 independent primary AML lines of each genotype to ABT-737 or S63845, either alone or in combination with the other drugs, at multiple concentrations, using high throughput robotics. Bliss analysis [33, 34] revealed significant synergies (positive scores) for certain drug combinations.

**Fig. 6:**
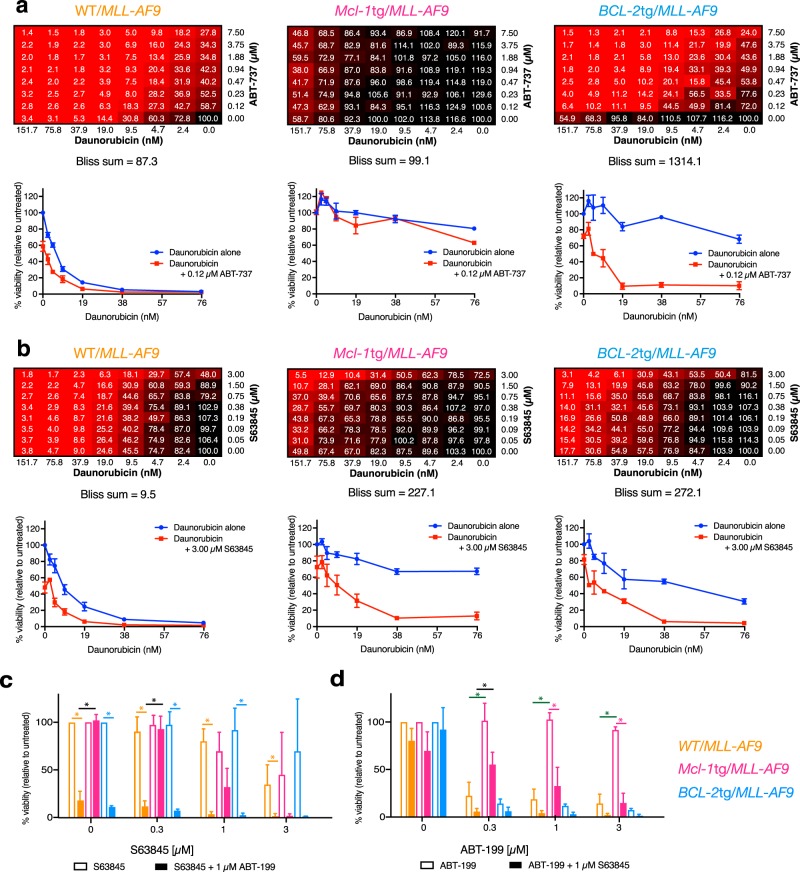
Synergy between daunorubicin and BH3 mimetics in vitro. Combination responses of daunorubicin plus **a** ABT-737 or **b** S63845 on WT/*MLL-AF9* (left), *Mcl-1*tg/*MLL-AF9* (middle) and *BCL-2*tg/*MLL-AF9* (right) primary AML cell lines as indicated by an 8 × 8 matrix informing on viability after 24 h treatment determined by CellTiter-Glo assay and the sum of Bliss scores across the combination dose matrix. The score for each dose combination is calculated according to the formula (A + B) − (A × B) where A and B are the fractional growth inhibitions induced individually by agents A and B at that given dose. Also shown is the response to daunorubicin across the dose range as a single agent and in combination with a single dose of (**a**) ABT-737 (0.12 µM) or (**b**) S63845 (3.00 µM). Each matrix is the average response of 3 independent tumours for each genotype. Viability curves indicate mean ± S.E.M of 3 independent tumours per genotype. Tumours used for treatment with daunorubicin + ABT-737 were WT/*MLL-AF9* #1560, #2036, #2037, *Mcl-1*tg/*MLL-AF9* #1554, #2038, #2039, *BCL-2*tg/*MLL-AF9* #1751, #2040, #2041. Tumours used for treatment with daunorubicin + S63845 were WT/*MLL-AF9* #1746, #1802, #1833, *Mcl-1*tg/*MLL-AF9* #1770, #1780, #1805, *BCL-2*tg/*MLL-AF9* #1751, #1765, #1807. **c, d** Responses to combination treatment with BH3 mimetics ABT-199 and S63845 were assessed by culturing primary AML cell lines for 24 h in varying concentrations of (**c**) S63845 ± 1 μM ABT-199 or (**d**) varying concentrations of ABT-199 ± 1μM S63845. Viability was determined via flow cytometric quantitation of annexin V^–^PI^–^ cells and expressed relative to untreated controls. Values are mean ± SEM of 3–4 independent tumour cell lines per genotype. *p < 0.05. Orange, pink and blue asterisks indicate significant difference between single agent and combination for that genotype. Black asterisk indicates significant difference between WT/*MLL-AF9* and *Mcl-1*tg/*MLL-AF9* combination treatments. Green asterisk indicates significant difference between WT/*MLL-AF9* and *Mcl-1*tg/*MLL-AF9* single agent treatments. Tumours used were WT/*MLL-AF9* #1744, #1802, #1833, #1834, *Mcl-1*tg/*MLL-AF9* #1750, #1770, #1780, *BCL-2*tg/*MLL-AF9* #1751, #1762, #1763

Figure 6a, b shows the outcome for daunorubicin. With the WT/*MLL-AF9* AMLs (left panels), inclusion of ABT-737 (Bliss 87.3) increased sensitivity more than inclusion of S63845 (Bliss 9.5). As anticipated, *BCL-2*tg/*MLL-AF9* AMLs (right panels) were more sensitive to daunorubicin plus ABT-737 (Bliss sum 1314.1) than to daunorubicin plus S63845 (Bliss sum 272.1) and, conversely, the *Mcl-1*tg/*MLL-AF9* AMLs (middle panels) were more sensitised by the MCL*-*1*-*specific BH3 mimetic (Bliss sum 227.1) than by ABT-737 (Bliss sum 99.1).

A similar pattern was noted with cytarabine, flavopiridol, and PIK-75 (Supplementary Figures S8, S9); the MCL-1 overexpressing AMLs were sensitised by S63845 to a greater extent than by ABT-737, and the BCL-2 overexpressing AMLs were sensitised more by ABT-737 than by S63845.

Notably, the AMLs also responded well to combining S63845 and ABT-199; any residual resistance to one BH3 mimetic at a particular concentration was significantly or completely overcome by including the other (Fig. 6c, d).

### Drug sensitivity of human AML cell lines

**Fig. 7:**
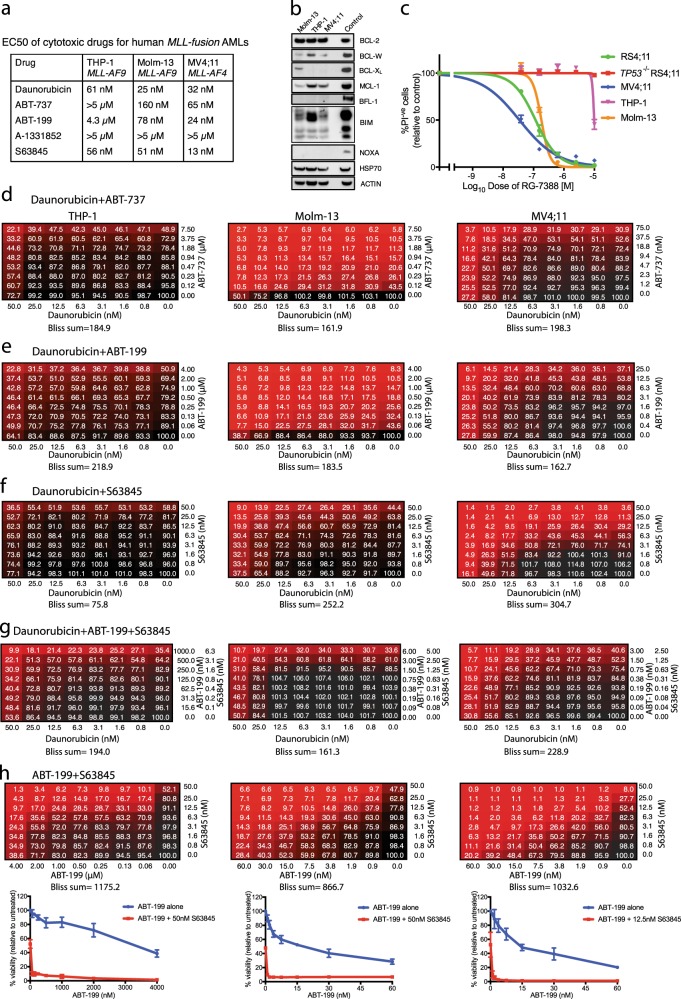
Response of human *MLL*-fusion AML cell lines to BH3 mimetics. **a** EC50s of daunorubicin and BH3 mimetics. The indicated cell lines were treated for 24 h with drug (0–10 μM) and viability was determined by CellTiter-Glo assay on a microplate reader. Values were calculated using GraphPad Prism by log-transforming the data and fitting it to a nonlinear regression. Data were determined from three independent experiments, each performed in triplicate. **b** Immunoblot analysis to detect indicated BCL-2 protein family members. Positive controls were lysates from the cell lines RS4;11 (for BFL-1) and U266 (for all other proteins). HSP 70 and ACTIN were used as loading controls. **c** Treatment of cell lines with MDM-2 inhibitor RG-7388 (0–10 μM) to determine p53 status [60]. Viability (PI-negative) was determined by flow cytometry. Data represent means ± SEM, determined from 3 independent experiments, each performed in triplicate. RS4;11 and p53^−/−^ RS4;11 cell lines were employed as controls. The data indicate that p53 is functional in MOLM-13 and MV4;11 but not in THP-1. **d**–**h** Combination responses of THP-1 (left), Molm-13 (middle) and MV4;11 (right) human *MLL-*fusion AMLs to (**d**) daunorubicin plus ABT-737, (**e**) daunorubicin plus ABT-199, (**f)** daunorubicin plus S63845, (**g**) daunorubicin plus ABT-199 + S63845, or (**h**) ABT-199 plus S63845. Drug concentrations are indicated on x and y-axes of the matrices. All lines were tested with 0–50 nM daunorubicin but the concentration range for the BH3 mimetics was varied according to the EC50 of the line under test. Cell viability was determined after 24 h by CellTiter-Glo assay and normalised to the viability of untreated samples. The sum of Bliss scores is shown below each matrix. The data represent means determined from three independent experiments. Also shown in (**h**) is the response to ABT-199 across the dose range as a single agent and in combination with S63845 (50 nM for THP-1 and Molm-13; 12.5 nM for MV4;11); data shown represent means ± SEM from three independent experiments

We also tested three human *MLL*-fusion AML cell lines: THP-1, MOLM-13 and MV4;11 [35–37] (Fig. 7). Each expresses BCL-2, BCL-W, and MCL-1, with BCL-X_L_ and BFL-1 being low or undetectable (Fig. 7b). EC50s were determined using CellTiter-Glo (Fig. 7a). THP-1 cells were the most resistant to daunorubicin, ABT-737 and ABT-199; and MV4;11 cells were the most sensitive to S63845. All lines were resistant to A-1331852, which was not tested further.

ABT-737, ABT-199 and S63845 were then tested in combination with daunorubicin (Fig. 7d–f); and ABT-199 plus S63845 was tested with or without daunorubicin (Fig. 7g, h). Each combination was significantly synergistic. Of note, Bliss scores of ~ 1000 were achieved for each human cell line by combining ABT-199 and S63845 (Fig. 7h). BH3 mimetics act downstream of p53 and, as predicted, the combination was effective even for THP-1 cells, which lack functional p53 (see Fig. 7c).

### In vivo tests of drug sensitivity

**Fig. 8:**
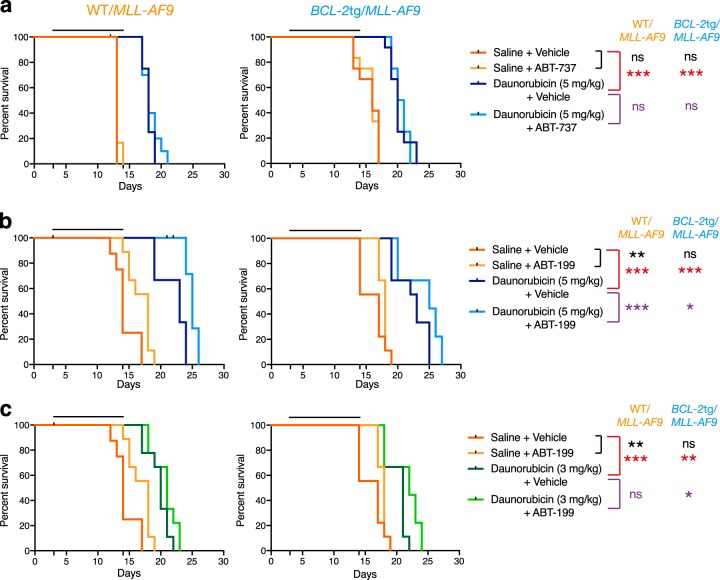
Synergy between daunorubicin and BH3 mimetics in vivo. Kaplan*–*Meier survival curves for mice transplanted with 0.5 × 10^6^ bone marrow cells from WT/*MLL-AF9* (left) or *BCL-2*tg/*MLL-AF9* (right) AMLs and treated with **a** 5 mg/kg daunorubicin ± 75 mg/kg ABT-737, **b** 5 mg/kg daunorubicin ± 100 mg/kg ABT-199 and **c** 3 mg/kg daunorubicin ± 100 mg/kg ABT-199. Daunorubicin was administered intravenously on days 1, 4 and 9; and ABT-737 or ABT-199 was administered intraperitoneally or by oral gavage respectively on days 1–5 and 8–12; controls received saline and ABT-737 vehicle. Transplanted mice were monitored daily for symptoms of AML and euthanased if morbidly ill. 3–4 independent tumours of each genotype were treated, with 3 mice per tumour per treatment arm. WT/*MLL-AF9* #1501, #1533, #2036 and #1742 (daunorubicin ± ABT*-*737 only), *BCL-2*tg/*MLL-AF9* #1537, #1563, #2028 and #1538 (daunorubicin ± ABT-737 only). Statistical significance between saline versus BH3 mimetic alone (black), saline vs daunorubicin alone (red) or daunorubicin vs daunorubicin + BH3 mimetic (purple) was determined by Log-rank (Mantle–Cox) test: ns = not significant, *p < 0.05, **p < 0.01, ***p < 0.001

To investigate whether ABT-737 could increase sensitivity to daunorubicin in vivo, bone marrow cells from 4 independent secondary WT/*MLL-AF9* AMLs and *BCL-2*tg/*MLL-AF9* AMLs were transplanted into non-irradiated syngeneic recipients and treatment was begun 3 days later (Fig. 8a). Although drug treatment extended the life of the animals transplanted with WT/*MLL-AF9* AMLs by 5 days, the combination of daunorubicin with ABT-737 was no more effective than daunorubicin alone. Comparable results were obtained for *BCL-2*tg/*MLL-AF9* AMLs.

Using the same AMLs, we also tested the efficacy of BCL-2-specific ABT-199 (Fig. 8b, c). ABT-199 had some efficacy as a single agent (compare curves for ABT*-*199 (yellow) and vehicle controls (pink)), probably because it could be used at a higher concentration (100 mg/kg) than ABT-737 (75 mg/kg) and has a somewhat higher affinity for BCL*-*2 [25]. Using the same dose of daunorubicin (5 mg/kg) as with ABT*-*737, combination therapy with ABT-199 was more efficacious than treatment with daunorubicin alone, for tumours of both genotypes (Fig. 8b; *p* < 0.001 for WT/*MLL-AF9* AMLs, *p* < 0.05 for *BCL-2*tg/*MLL-AF9* AMLs), although all treated mice still died within 30 days. The benefit of combination therapy with ABT*-*199 was still apparent at a lower dose of daunorubicin (3 mg/kg) for *BCL-2*tg/*MLL-AF9* AMLs (*p* < 0.05) although not for WT/*MLL-AF9* AMLs (Fig. 8c).

We were unable to use S63845 at high enough concentrations to effectively test its efficacy in vivo against the *Mcl-1*tg/*MLL-AF9* AMLs, which overexpress mouse MCL-1.

## Discussion

AML remains a major clinical challenge [32], with little improvement in patient outcomes over the past 30 years. The high levels of pro-survival proteins BCL-2 and MCL-1 commonly found in AMLs [11, 12, 38], especially after relapse [14], are thought to contribute to resistance to therapy. In this study, we directly tested the impact of MCL-1 and BCL-2 using the well-studied *MLL-AF9* mouse model.

### Impact on AML development

Elevated expression of BCL-2 or MCL-1 had no significant impact on the kinetics of morbidity of *MLL-AF9*-driven AML (Fig. 1d), in contrast to *Myc*-driven B lymphoid malignancy, which was markedly accelerated by *BCL-2* or *Mcl-1* transgenes [21, 39]. However, the much faster tumour development in the AML model permits little scope for acceleration.

There were, nonetheless, intriguing differences between WT/*MLL-AF9* AMLs and those overexpressing BCL-2 or MCL-1. Leukocytosis became apparent earlier and was more severe in the *BCL-2*tg/*MLL-AF9* and *Mcl-1*tg/*MLL-AF9* mice (Fig. 1) and the AML phenotype was more differentiated, as were *MLL-AF9* AMLs lacking the pro-apoptotic BH3-only protein BIM [22] (Fig. 2 and Supplementary Figure S4, S5). These observations suggest that blocking apoptosis enhances the in vivo lifespan of maturing *MLL-AF9-*driven leucocytes. However, in contrast to results for a mouse model of the myelodysplastic syndrome [40], leukaemic transformation was unaffected, as the *BCL-2*tg/*MLL-AF9* and *Mcl-1*tg/*MLL-AF9* AMLs were fully and serially transplantable in non-irradiated mice (Supplementary Table S3).

Transgenic overexpression of MCL-1 and BCL-2 appeared to modulate the expression pattern of other BCL-2 family members (Fig. 3). Of note, higher levels of BIM, BMF and NOXA were apparent in *Mcl-1*tg/*MLL-AF9* and *BCL-2*tg/*MLL-AF9* AMLs than in WT/*MLL-AF9* AMLs, suggesting that elevated pro-survival proteins had preserved *MLL-AF9*-driven cells experiencing stress-induced upregulation of these pro-apoptotic BH3-only proteins. Furthermore, the *BCL-2*tg/*MLL-AF9* AMLs had lower levels of endogenous BCL-2 and BCL-X_L_ than the WT/*MLL-AF9* AMLs, suggestive of lower selection pressure to upregulate these endogenous pro-survival proteins. Comparable changes were less apparent for the *Mcl-1*tg/*MLL-AF9* AMLs, probably because the level of transgenic MCL-1 protein is not as high as that of transgenic BCL-2 [41], although five of eight had lower endogenous BCL-2 than WT/*MLL-AF9* AMLs. Overall, these results suggest that *MLL-AF9* expression imposes considerable cytotoxic stress on haemopoietic cells, either directly or indirectly, and this selects for upregulation of pro-survival proteins. Genetic studies have shown that endogenous MCL-1 is essential for the development and sustained expansion of AML driven by MLL-fusion genes, whereas BCL-X_L_, BCL-2 and BCL*-*W play lesser roles [15].

### Impact on drug sensitivity

In vitro tests of drug sensitivity were performed using short-term cell lines derived from five primary AMLs of each genotype (Fig. 4, Supplementary Figure S5). Four classes of drugs were used: cytotoxic drugs routinely used to treat AML (daunorubicin, cytarabine and etoposide); the proteasome inhibitor bortezomib, which is being trialled for AML; [19] CDK7/9 inhibitors that inhibit transcription and thereby reduce levels of short-lived proteins such as MCL-1; [16, 17, 42] and BH3 mimetics targeting different pro-survival BCL-2 family members: ABT-737 (which targets BCL-2, BCL*-*X_L_ and BCL-W) [24, 28], ABT-199 (venetoclax) (specific for BCL-2) [25], A-1331852 (specific for BCL-X_L_) [34, 43] and S63845 (specific for MCL-1) [26].

The WT/*MLL-AF9* AMLs were far more sensitive to daunorubicin (EC50 5.9 nM) than to etoposide or cytarabine (EC50s of 200 nM and 530 nM respectively) and, as predicted, overexpression of MCL-1 or BCL-2 increased resistance (Fig. 4). MCL*-*1 was as inhibitory as BCL-2, which suggests it is more potent, given the lower level of the transgenic protein [41]. Our gene knock*-*out studies have previously shown that responsiveness to daunorubicin, etoposide and cytarabine is dependent on the BH3-only proteins PUMA and NOXA and that BIM also plays a role in responsiveness to cytarabine [22].

PIK-75, a dual inhibitor of CDK9 and PI3K, has been shown to be active against cytologically diverse primary human AML samples [17]. However, neither PIK-75 nor the other CDK7/9 inhibitors were as cytotoxic as daunorubicin against the mouse *MLL-AF9* AMLs, despite reducing MCL-1 protein levels in a dose*-*dependent manner, even in the *Mcl-1*tg/*MLL-AF9* tumours.

In contrast, bortezomib was very potent, even in the face of high BCL-2 or MCL-1. Our previous study [22] showed that NOXA is very important for bortezomib-mediated killing of *MLL-AF9* AMLs. NOXA levels are high in most *MLL-AF9* AMLs we tested, particularly those overexpressing MCL-1 or BCL-2 (Fig. 3). NOXA strongly binds to and inhibits pro-survival MCL-1 [44, 45], which is vital for the development and sustained growth of AMLs driven by *MLL-*fusion genes [15]. Presumably NOXA levels are sufficiently elevated by bortezomib to overcome MCL-1 even in the *Mcl-1*tg/*MLL-AF9* AMLs.

BH3 mimetics bind tightly to the surface groove of their target pro-survival proteins, thereby preventing them from binding to BH3-only proteins and to activated BAX and/or BAK molecules (see [10, 46] and references therein). In doing so, the BH3 mimetics increase the availability of BH3-only proteins to carry out their death-promoting functions. As single agents, the BH3 mimetics were not highly potent against WT/*MLL-AF9* AMLs, BCL-X_L_-specific A-1331852 being the least effective. Overexpression of MCL-1 dramatically increased resistance to ABT-737 and ABT-199 but, importantly, BCL-2 overexpressing lines were as sensitive as WT/*MLL-AF9* AMLs (Fig. 4). Conversely, overexpression of BCL-2 increased resistance to the MCL*-*1*-*specific S63845, but overexpression of MCL-1 did not.

Of note, when ABT-737 and S63845 were tested in vitro in combination with the other cytotoxics (Fig. 6, Supplementary Figures S8, S9), significant synergies could be achieved. With daunorubicin, those overexpressing MCL-1 were more sensitised by the MCL*-*1-specific S63845 (Bliss sum 227.1) than by ABT-737 (Bliss sum 99.1), and those overexpressing BCL-2 were more sensitised by the BCL-2-binding compound ABT*-*737 (Bliss sum 1314), although S63845 also had clear impact (Bliss sum 272.1) (Fig. 6). In view of the approximately 6-fold greater affinity of S63845 for human versus mouse MCL-1 [26], these results predict that S63845 would exhibit even greater synergy in human cells, as was demonstrated for three MLL-fusion AMLs (Fig. 7).

To investigate whether synergy with daunorubicin extended to treatment in vivo, we transplanted multiple independent WT/*MLL-AF9* and *BCL-2*tg/*MLL-AF9* AMLs into immunocompetent recipients, which were then treated with: ABT-737 or ABT-199 alone, daunorubicin alone, ABT-737 or ABT-199 plus daunorubicin, or no drug (Fig. 7). ABT-199 showed modest efficacy as a single agent for the BCL-2 overexpressing AMLs, whereas ABT-737 did not, probably because the dosage had to be lower and ABT-199 has somewhat higher affinity for BCL-2 [25]. Mouse survival increased significantly when ABT-199 was combined with daunorubicin, for both the WT/*MLL-AF9* and *BCL-2*tg/*MLL-AF9* AMLs. Pertinently, a recent in vitro study of primary human AML cells found that venetoclax synergised with another anthracycline (idarubicin) and also cytarabine [13].

Looking forward, while BH3 mimetics and other targeted therapies seem unlikely to replace standard chemotherapeutics for AMLs, their combination with current regimens may significantly improve outcomes. For AMLs expressing both BCL-2 and MCL-1 it could be advantageous to include agents targeting both BCL-2 and MCL-1, should this prove tolerable.

Venetoclax (ABT-199), approved for use in refractory chronic lymphocytic leukaemia, is being trialled in combination therapies for other lymphoid leukaemias and lymphomas [10, 47]. Venetoclax and S63845 were recently shown to have encouraging efficacy against primary human AML cells in vitro; some AMLs were responsive to both, others only to one or the other [26]. Promising results have also been reported for venetoclax as monotherapy in a phase II trial for relapsed or refractory AML [48] and emerging preclinical and clinical data suggest that venetoclax combination therapy may significantly improve outcomes [13, 49–53]. Accordingly, venetoclax has received Breakthrough Therapy Designation for treatment*-*naïve AML patients ineligible to receive high dose chemotherapy.

Phase 1 clinical trials are just beginning for S63845 and two other recently reported MCL-1-specific BH3 mimetics. Valid safety concerns have been raised by reports of MCL-1 dependence for mouse cardiomyocytes [54], hepatocytes [55] and neurons [56]. Nevertheless, *Mcl-1*^+/-^ mice, which model the impact of permanent 50% inhibition of MCL-1, are normal and healthy [57], suggesting that a suitable therapeutic window may well be found, especially for intermittent treatment.

## Materials and methods

### AML generation

AMLs were generated by *MLL-AF9* retroviral transduction of haemopoietic stem/progenitor cells (HSPC) and reconstitution of sublethally irradiated C57BL/6*-*Ly5.1 mice, as described [5, 58] except that E14.5 foetal liver was the source of HSPC rather than bone marrow [22]. Mice were monitored regularly and euthanased when showing severe AML symptoms (hunched stance, ruffled coat, lethargy, anaemia and splenomegaly) or significant weight loss. Histological and haemopoietic analysis is detailed in the Supplementary Methods. Mouse survival analysis utilised GraphPad Prism and significance was determined using Log-rank (Mantel–Cox) test.

### Cell lines

Human *MLL*-fusion AML cell lines MV4;11 [37] and THP-1 [35] were from ATCC (American Type Culture Collection; VI, USA) and Molm-13 [36] from DSMZ (Deutsche Sammlung von Mikroorganismen und Zellkulturen; Leibniz, Germany). Culture medium was RPMI 1640 + 10% FBS, with inclusion of 50 μM 2-mercaptoethanol for THP-1 cells.

### In vitro drug treatment

Short-term *MLL-AF9* AML lines were generated as described [22] and drug sensitivity was determined by flow cytometric analysis of apoptosis or high throughput CellTiter-Glo (Promega, Madison WI, USA) measurement of metabolic activity (see Supplementary Methods). Drug sensitivity of human *MLL-*fusion AML cell lines was determined by CellTiter-Glo assay on a microplate reader (LUMIstar OPTIMA, Germany).

### In vivo drug treatment

Primary tumours were expanded in vivo and transplanted into non-irradiated recipient mice as previously described [22] to generate secondary tumours, which developed after 13–50 d. Drug treatment of transplanted secondary tumours was performed as published [22, 59]. Briefly, this involved intravenous injection of daunorubicin (3 mg/kg or 5 mg/kg) or an equal volume of saline on days 1, 4 and 9, followed by a flush of saline, and either ABT*-*737 (75 mg/kg injected intraperitoneally) or ABT-199 (100 mg/kg administered by oral gavage), or equal volume of vehicle, on days 1 to 5 and days 8 to 12. Mice were euthanased at ethical endpoint.

See Supplementary Materials and Methods for mice, haemopoietic analysis, drug treatment and western blotting.

## Electronic supplementary material

Supplementary Information
