# Supplementary material for: Impact of elevated anti-apoptotic MCL-1 and BCL-2 on the development and treatment of MLL-AF9 AML in mice
Source: Cell Death Differ. 2018 Nov 23;26(7):1316–31. doi: 10.1038/s41418-018-0209-1 (PMC6748137; doi:10.1038/s41418-018-0209-1)
Supplement: Supplementary file 1 — Supplementary Information [file 41418_2018_209_MOESM1_ESM.pdf]

Anstee et al Supplementary Information

Supplementary Materials and Methods

Mice. VavP-*Mcl-1*(33) transgenic (hereafter *Mcl-1*tg)[1] and vavP-*BCL-2*(69) transgenic (hereafter *BCL-2*tg) mice[2] were generated and maintained on a C57BL/6 (Ly5.2) background at the Walter and Eliza Hall Institute (WEHI) and genotyped as described.[1,2] Experimental protocols were approved by WEHI's Animal Ethics Committee.

Human cell lines. MV4;11 and THP-1 were obtained from ATCC (American Type Culture Collection) and Molm-13 from DSMZ (Deutsche Sammlung von Mikroorganismen und Zellkulturen). Culture medium was RPMI 1640 containing 10% FBS, with inclusion of 50 μM 2-mercaptoethanol for THP-1 cells.

Haemopoietic analysis. Healthy and sick mice were analysed similarly. Blood was collected via retro-orbital bleed or cardiac puncture. Single-cell suspensions were prepared from spleen and bone marrow (flushed from femurs and tibias). Red blood cells were removed from blood and spleen using 0.168 M ammonium chloride. Peripheral blood cell counts were enumerated using an ADVIA 2120 analyser (Siemens, Erlangen, Germany) and organ cell counts enumerated on a Casy Counter (Scharfe, Reutlingen, Germany). Cell composition was determined by immunostaining and flow cytometry (LSR I flow cytometer, BD Biosciences, Franklin Lakes NJ, USA), using FlowJo Version 9.3.2 (TreeStar, Ashland, OR, USA). Monoclonal antibodies against cell surface markers were produced and labelled (in-house) with biotin, fluorescein isothiocyanate (FITC), R-phycoerythrin (PE) or allophycocyanin (APC). Antibodies used included: RB6-8C5, anti-Gr1; MI/70, anti-Mac1; A201.1, anti-CD45.1; 5.405.125.2, anti-CD45.2; anti-F4/80; RA3-6B2, anti-B220; 5.1, anti-IgM; 1145-2C11, anti-IgD; YTA3.2.1, anti-CD4; 53.6.7.2, anti-CD8; ACK4, anti-cKit.

Histological analysis. A combination of lymph node, spleen, pancreas, kidney, liver, ovary, lung, heart, and/or sternum were collected and fixed in 10% formalin. The samples were embedded in paraffin wax and stained with hematoxylin and eosin by WEHI's histology department. For blood films, 2 μL of blood was smeared onto a microscope slide and left to dry overnight. Slides were then stained with May-Grünwald Giemsa. For bone marrow cytospins, cells were diluted in PBS and 1-5x10^4 cells in 200 μL PBS was loaded into a

cytospin cassette. Centrifugation was performed in a Cytospin 3 centrifuge (Thermo Shandon Ltd, Cheshire, UK) at 113 x *g* for 5 min with low acceleration. The slides were left to air dry overnight then stained with May-Grünwald Giemsa. Sections and films were assessed (blinded as to genotype) using an Olympus BX43 microscope (Olympus, Tokyo, Japan); photographs were taken with an Olympus DP72 camera.

Drug treatment. Drugs used in *in vitro* assays or *in vivo* treatments were: etoposide (Pfizer, New York City, NY, USA); daunorubicin (Pfizer); cytarabine (Sigma-Aldrich, St. Louis, MO, USA); bortezomib (LC Laboratories, Woburn, MA, USA); flavopiridol (Selleck Chemicals, Houston, TX, USA); PIK-75 (Selleck Chemicals); SNS-032 (Selleck Chemicals); dinaciclib (Selleck Chemicals); ABT-737 (Active Biochem, Hong Kong, China); ABT-199 (Active Biochem, Hongkong); S63845 (Synthesis MedChem, Victoria, Australia and Active Biochem, Hong Kong); A1131852 (WEHI, Victoria, Australia); idasanutlin RG7388 (Chemgood, VA USA).

Drug sensitivity was determined by flow cytometric analysis of apoptosis or high throughput CellTiter-Glo luminescence (Promega, Madison WI, USA) measurement of metabolic activity. For the former, cells were seeded at $1x10^5$ cells per well in 96 well plates and incubated for 24 h in IMDM medium (Gibco, Waltham, MA, USA) containing 10% FCS (Gibco) and 6 ng/mL recombinant IL-3 (WEHI cytokine service, Victoria, Australia) and the test drugs, after which annexin V-Alexa647 (made in house) and 4 µg/mL propidium iodide (Sigma-Aldrich) was added and viability (annexin V^-PI^-) assessed using a FACSCalibur (BD Biosciences, Franklin Lakes NJ, USA). For the high-throughput CellTiter-Glo assay, a Microlab STAR Line (Hamilton, Reno, NV, USA) workstation was used to cross-titrate compounds in an 8x8 dose matrix diluted in 2-fold steps. 1000 cells in 40 µL IMDM supplemented with 10% FCS and 6 ng/mL recombinant IL-3 were seeded per well in a 384 well plate using a Multidrop™ Combi Reagent dispenser (ThermoFisher Scientific, Waltham MA, USA) and compounds added using the 100 nL pin tool. Cells were incubated at 37°C for 24 h before assessing viability using 25 µL CellTiter-Glo reagent according to the manufacturer's instructions.

Bliss scores. Bliss scores were calculated for each drug combination in the indicated dose matrix using the formula (A+B) - (AxB) where A and B are the fractional growth inhibitions induced individually by agents A and B at that given dose and totalled to give a "Bliss sum"

value;[3,4] negative integers indicate antagonism, zero indicates additive activity and positive integers indicate synergy.

Western blotting. Bcl-2 family protein and p53 expression was analysed pre- and post-treatment. To prevent cell death post-treatment, 25 μM Q-VD-OPh (MP Biochemicals, Santa Ana, CA, USA) was added to 2x10^6 primary *MLL-AF9* tumour cells and cells were then plated in a 6 well plate in a volume of 2 mL IMDM supplemented with 10% FCS and 6 ng/mL recombinant IL-3. Cells were treated *in vitro* with PIK-75 (Selleck Chemicals, Houston, TX, USA), flavopiridol (Selleck Chemicals), SNS-032 (Selleck Chemicals) or bortezomib (LC Laboratories, Woburn, MA, USA) at indicated concentrations for 6 h, then harvested and frozen at -80°C until lysates were made for western blotting. Lysates were made from cell pellets using RIPA buffer containing a cOmplete™ protease inhibitors (Roche, Basel, Switzerland) and 1 mM phenylmethylsulfonyl fluoride (PMSF) (Sigma-Aldrich, St. Louis, MO, USA). Proteins (25 μg) were resolved by electrophoresis on SDS-PAGE (pre-cast 10% Bis-Tris NuPage) gels (Life Technologies, Carlsbad, CA, USA) using MES Running buffer (Life Technologies) then transferred onto nitrocellulose membranes (iBlot, Life Technologies). Membranes were blocked with 5% non-fat dry milk (Devondale, Victoria, Australia) in MT-PBS with 0.1% Tween-20 (Sigma-Aldrich) (PBS-T) and then probed with antibodies for A1/BFL-1 (clone 6D7, WEHI mAb lab; Cell Signaling #D1A1C), BCL-W (rabbit polyclonal, WEHI mAb lab), β-ACTIN (clone AC-74, Sigma, catalog #A2228), BAD (Enzo Life Sciences, catalog #IMG-5665), BAK (clone DF9, WEHI mAb lab), BAX (clone 49F9-13-3, WEHI mAb lab), human BCL-2 (clone Bcl-2-100, WEHI mAb lab), mouse BCL-2 (clone 3F11, WEHI mAb lab), BCL-X_L (BD Biosciences, catalog #556361), BID (clone 2D1-3, WEHI mAb lab), BIM (Enzo Life Sciences, catalog #ADI-AAP-330E; Stressgen rabbit polyclonal), BMF (clone 17A9, WEHI mAb lab), HSP-70 (clone N6, W Welch USCF), MCL-1 (clone 19C4-15, WEHI mAb lab), NOXA (ProSci, catalog #2437), p53 (Novocastra, catalog #CM5) and PUMA (ProSci, catalog #3043). Blots were then incubated with HRP-conjugated secondary antibodies (Southern Biotech, Birmingham, AL, USA), and visualised using LuminataTM Forte western HRP substrate (Merck-Millipore, Billerica, MA, USA) on a ChemiDocTM XRS+ Molecular Imager® (Bio-Rad, Hercules, CA, USA). Prior to re-probing, blots were incubated with 0.1% 1 M sodium azide in PBS-T or stripping buffer containing 0.5 mM TrisHCl (Life Technologies), 10% SDS (Amresco, Solon, OH, USA) and 100 mM 2-mercaptoethanol (Sigma-Aldrich) to remove signal of previous antibody.

Supplementary Figures

Figure S1 Overexpression of MCL-1 or BCL-2 provokes early accumulation of donor-derived myeloid cells in mice reconstituted with *MLL-AF9* virus-infected foetal liver cells. 3 wk analysis of haemopoietic tissues in mice reconstituted with WT/GFP (light orange, n=7-18), *Mcl-1*tg/GFP (light pink, n = 6-19), *BCL-2*tg/GFP (light blue, n = 8-22), WT/*MLL-AF9* (orange, n = 5-27), *Mcl-1*tg/*MLL-AF9* (pink, n = 7-26) or *BCL-2*tg/*MLL-AF9* (blue, n = 6-25) cells. **(a)** Percentage of donor-derived cells indicated by $Ly5.2^+GFP^+$ cells in the spleen, bone marrow and blood. **(b)** Percentage of $Mac1^+$ cells within the population of donor cells ($Ly5.2^+GFP^+$) in each organ. Data represents mean ± S.E.M. * $p<0.05$, ** $p<0.01$, *** $p<0.001$, calculated by Student's T test with Welch's correction. See also Table S1.

Figure S2 *MLL-AF9* expression causes myeloid leukaemia. Flow cytometric analysis of spleen, bone marrow and blood of healthy control mice [reconstituted with WT/GFP (light orange, n = 8), *Mcl-1*tg/GFP (light pink, n = 9), *BCL-2*tg/GFP (light blue, n = 8-9) cells] and sick *MLL-AF9* AML mice at autopsy [reconstituted with WT/*MLL-AF9* (orange, n = 10-13), *Mcl-1*tg/*MLL-AF9* (pink, n = 13) or *BCL-2*tg/*MLL-AF9* (blue, n = 9-13) cells]. **(a, b)** Percentage of donor-derived cells is indicated by **(a)** $Ly5.2^+GFP^+$ cells or **(b)** Ly5.1 negative cells. **(c)** Percentage of $Mac1^+$ cells within $Ly5.2^+GFP^+$ population. Data represent mean ± S.E.M. **(d)** FACS plots of bone marrow cells from three sick *Mcl-1*tg/*MLL-AF9* mice (#1780, #1804 and #1854) gated on GFP^+ cells showing variability in the proportion of $Mac1^+$ and $Gr1^+$ cells. **(e)** Percentage of $Mac1^+$ single positive (SP) and $Mac1^+Gr1^+$ double positive (DP) cells, when gated on GFP^+ cells, in spleen, bone marrow and blood of sick mice that had been reconstituted with WT/*MLL-AF9* (orange; n = 12-14), *Mcl-1*tg/*MLL-AF9* (pink; n = 15) or *BCL-2*tg/*MLL-AF9* (blue; n = 12-15) cells. Each dot represents an individual mouse and mean ± S.E.M is indicated. ** $p<0.01$, *** $p<0.001$, calculated by Student's T test with Welch's correction.

Figure S3 Histological comparison of sick *MLL-AF9* AML and healthy control mice. **(a)** Sections of spleen, kidney, liver, pancreas and bone marrow stained with hematoxylin and eosin (H&E) of healthy WT/GFP control mouse compared to representative sick WT/*MLL-AF9* (tumour #1834), *Mcl-1*tg/*MLL-AF9* (tumour #1804) and *BCL-2*tg/*MLL-AF9* (tumour #1836) mice. Scale bars: kidney, liver, spleen and pancreas 500 μm, BM 50 μm, BM

(insert) 20 μm. **(b)** Arbitrary scores based on degree of infiltration (performed blinded) of kidney, liver and pancreas. H&E sections were assessed for WT/*MLL-AF9* (orange, n = 11-12), *Mcl-1*tg/*MLL-AF9* (pink, n = 8-10) or *BCL-2*tg/*MLL-AF9* (blue, n = 6) mice. Each dot represents one mouse and mean ± S.E.M is indicated. Black ringed dots indicate mice shown in **(a)**.

Figure S4 Leukocytosis in sick primary AML mice. **(a)** Representative blood smears from sick mice of the following genotypes: WT/*MLL-AF9* (tumours #1832 and #2025), *Mcl-1*tg/*MLL-AF9* (tumours #1854 and #2026), *BCL-2*tg/*MLL-AF9* (tumours #1836 and #2029) and $Bim^{-/-}$/*MLL-AF9* (tumours #1157 and #1158). Scale Bars: 20 μM, 1000x magnification. **(b)** Differential counts of blood leukocytes in 6-14 sick mice of each genotype. **(c)** Representative bone marrow cytospins of the following genotypes: WT/*MLL-AF9* (tumours #1533 #2025), *Mcl-1*tg/*MLL-AF9* (tumours #1805, #2026) and *BCL-2*tg/*MLL-AF9* (tumours #1806, #1856), $Bim^{-/-}$/*MLL-AF9* (tumours #1157, #1249). Scale Bars: 20 μM, 1000x magnification. **(d)** Differential counts of myeloid cell types in bone marrow of 4-9 mice of each genotype. Each dot represents one mouse and mean ± S.E.M is indicated. * p<0.05, ** p<0.01, *** p<0.001, calculated by Student's T test with Welch's correction.

Figure S5 Differential counts of primary, secondary and tertiary *MLL-AF9* AMLs. Bone marrow cytospins from primary (a), secondary (b) and tertiary (c) tumour-bearing mice were stained with May-Grünwald Giemsa and scored (blinded) for morphology by haematologist APN. Individual data points are presented as mean ± SD. Secondary AMLs were generated by injecting 2 x 10^6 spleen cells from primary AML-bearing mice into immunocompetent syngeneic secondary recipients via the tail vein. Tertiary AMLs were generated by injecting 0.5 x 10^6 bone marrow cells from secondary AML-bearing mice into immunocompetent syngeneic tertiary recipients (See Table S3 for transplantation efficiency). Two way ANOVA analysis used to determine significance of differences between WT/*MLL-AF9* and either *Mcl-1*tg/*MLL-AF9* or *Bcl2*tg/*MLL-AF9.* * indicates p value < 0.05, *** < 0.001, **** <0.0001.

Figure S6 Drug sensitivity of cultured primary *MLL-AF9* AMLs. Dose response of WT/*MLL-AF9* (orange), *Mcl-1*tg/*MLL-AF9* (pink) and *BCL-2*tg/*MLL-AF9* (blue) primary AML cell lines treated for 24 h with **(a)** chemotherapeutics etoposide, daunorubicin and

cytarabine; proteasome inhibitor bortezomib; CDK7/9 inhibitors flavopiridol, PIK-75, SNS-032 and dinaciclib or **(b)** BH3 mimetics ABT-737 (anti-BCL-2, Bcl-X_L, BCL-W), ABT-199 (anti-BCL-2), and S63845 (anti-MCL-1). **(c-e)** Similarly, short-term (**c**) WT/*MLL-AF9*, (**d**) *Mcl-1*tg/*MLL-AF9* and (**e**) *BCL-2*tg/*MLL-AF9* primary AML cell lines were treated with chemotherapeutics and BH3 mimetics, as indicated, in the absence (solid bar) and presence (empty bar) of 25 μM Q-VD-OPH (QVD) for 24 h. Viability was measured by flow cytometric quantitation of annexin V^- PI^- cells, expressed relative to untreated controls. 2-6 technical replicates per independent tumour were averaged. Data plotted are average response of 5-11 independent tumours per genotype ± S.E.M. Not all tumours were treated with all drugs. * $p<0.05$, calculated by Student's T test with Welch's correction (pink asterisk indicates significant difference between WT/*MLL-AF9* versus *Mcl-1*tg/*MLL-AF9*; blue asterisk indicates significant difference between WT/*MLL-AF9* versus *BCL-2*tg/*MLL-AF9*; black asterisk indicates significant difference between *Mcl-1*tg/*MLL-AF9* versus *BCL-2*tg/*MLL-AF9*). WT/*MLL-AF9* tumours: #1501, #1502, #1503, #1533, #1559, #1742, #1744, #1746, #1802, #1833, #1834, #2036. *Mcl-1*tg/*MLL-AF9* tumours: #1532, #1535, #1536, #1648, #1748, #1750, #1766, #1770, #1780, #1805, #1830, #1854, #2038. *BCL-2*tg/*MLL-AF9* tumours: #1563, #1647, #1751, #1762, #1763, #1764, #1765, #1806, #1807, #1835, #2040.

Figure S7 Impact of CDK inhibitors and bortezomib on apoptosis regulators in primary AML cell lines. Western blot analysis of expression of BCL-2 family proteins in cell lines derived from primary AMLs: WT/*MLL-AF9* (tumour #1559 left panel), *Mcl-1*tg/*MLL-AF9* (tumour #1766; central panel) and *BCL-2*tg/*MLL-AF9* (tumour #1806; right panel). Cells were incubated for 6 h with no drug, Q-VD-OPh (QVD) only or QVD with addition of PIK-75 (P), flavopiridol (F), SNS-032 (S) or bortezomib (B) at doses indicated (in μM for CDK inhibitors and nM for bortezomib). Molecular weight markers are indicated (kD).

Figure S8 Testing synergy between ABT-737 and other drugs. Viability of WT/*MLL-AF9* (left), *Mcl-1*tg/*MLL-AF9* (middle) and *BCL-2*tg/*MLL-AF9* (right) primary AML cell lines treated with ABT-737 plus one of cytarabine, bortezomib, flavopiridol, dinaciclib or PIK-75. Combination responses indicated by 8x8 matrix informing on viability after 24 h treatment determined by CellTiter-Glo assay. Each matrix is the average response of 3 independent tumours for each genotype. The sum of Bliss scores across the combination dose matrix is listed for each treatment condition.

Figure S9 Testing synergy between S63845 and other drugs. Viability of WT/*MLL-AF9* (left), *Mcl-1*tg/*MLL-AF9* (middle) and *BCL-2*tg/*MLL-AF9* (right) primary AML cell lines treated with S63845 plus one of cytarabine, bortezomib, flavopiridol, dinaciclib or PIK-75. Combination responses indicated by 8x8 matrix informing on viability after 24 h treatment determined by CellTiter-Glo assay. Each matrix is the average response of 3 independent tumours for each genotype. The sum of Bliss scores across the combination dose matrix is listed for each treatment condition.

Supplementary Table S1 Haemopoietic composition of mice 3 weeks post reconstitution

Organ/cell type	WT/GFP	*Mcl-1*tg/GFP	*BCL-2*tg/GFP	WT/*MLL-AF9*	*Mcl-1*tg/*MLL-AF9*	*BCL-2*tg/*MLL-AF9*
**Spleen**						
**Total Cells**	**133±90**	**124±84**	**151±67**	**148±54**	**291±174**	**290±216**
CD4^{+}CD8^{-}	14.9±7.2	12.8±8.3	11.5±4.8	12.3±8.2	14.4±6.0	12.5±4.5
CD4^{-}CD8^{+}	9.19±4.46	7.60±4.18	7.31±3.40	7.02±4.92	7.84±3.93	6.58±2.66
B220^{+}IgM^{-}/IgD^{-}	3.82±6.27	8.13±8.94	12.9±6.4^	3.59±2.66	7.87±4.80	13.6±9.6
B220^{+}IgM^{+}/IgD^{+}	20.4±11.9	45.6±34.7	84.3±43.7^^	14.8±6.9	35.3±16.8°	63.2±23.0°°
Mac1^{+}	3.66±2.03	4.84±4.33	5.15±2.33	12.4±8.7	26.2±22.4*	50.0±58.2
Mac1^{+}Gr1^{+}	12.8±8.3	9.99±9.00	6.46±3.13	55.2±26.5*	157±129*	122±156
**BM**						
**Total Cells**	**51.4±10.9**	**66.3±11.6^**	**75.9±13.6^^**	**72.2±10.6****	**88.8±17.2***	**86.6±21.2**
B220^{+}IgM^{-}/IgD^{-}	2.89±1.97	9.09±6.63	18.4±6.37^^^	2.05±1.31	2.44±1.23	6.90±6.69
B220^{+}IgM^{+}/IgD^{+}	0.811±0.543	3.51±3.99	8.73±7.39^	0.684±0.446	0.571±0.345	2.03±2.21*
Mac1^{+}	1.74±0.470	2.18±0.499	5.26±6.55	14.8±9.67*	16.4±13.9*	22.4±16.9
Mac1^{+}Gr1^{+}	14.4±4.00	19.6±6.30	21.6±11.1	33.7±13.7*	48.4±16.2**	43.5±18.2*
**Peripheral Blood**						
**Total Cells**	**1.83±0.71**	**2.19±1.19**	**4.57±5.71^**	**11.4±8.3*****	**67.3±66.8***°°°**	**120±148***°°**
Mac1^{+}	0.23±0.20	0.21±0.20	0.25±0.29	1.45±1.31***	15.2±18.9***°°	32.7±38.6***°°°
Mac1^{+}Gr1^{+}	0.28±0.19	0.28±0.18	0.35±0.26	6.90±6.25***	41.4±47.5***°°	61.6±88.0**°°
CD4^{+}CD8^{-}	nd	nd	nd	nd	nd	nd
CD4^{-}CD8^{+}	nd	nd	nd	nd	nd	nd
B220^{+}IgM^{-}/IgD^{-}	nd	nd	nd	nd	nd	nd
B220^{+}IgM^{+}/IgD^{+}	nd	nd	nd	nd	nd	nd

Nucleated cells × 10^{6}, except peripheral blood cells (× 10^{6}/mL) from mice 3 wk post reconstitution
n = 5-8 mice per genotype for spleen and BM and n=17-27 mice per genotype for peripheral blood.
Student's T-test with Welch's correction:
Significantly different from GFP of same genotype: ***p≤0.001; **p≤0.01; *p≤0.05
Significantly different from WT/GFP (only calculated for *Mcl-1*tg/GFP and *BCL-2*tg/GFP): ^^^p≤0.001; ^^p≤0.01; ^p≤0.05
Significantly different from WT/*MLL-AF9* (only calculated for *Mcl-1*tg/*MLL-AF9* and *BCL-2*tg/*MLL-AF9*): °°°p≤0.001; °°p≤0.01; °p≤0.05

Supplementary Table S2 Haemopoietic composition of sick *MLL-AF9* AML mice and healthy control mice

Organ/cell type	WT/GFP	*Mcl-1*tg/GFP	*BCL-2*tg/GFP	WT/*MLL-AF9*	*Mcl-1*tg/*MLL-AF9*	*BCL-2*tg/*MLL-AF9*
**Spleen**						
**Total cells**	**74.7±36.6**	**156±200**	**104±33**	**501±146*****	**730±198*****°°°	**724±159*****°°°
$CD4^+CD8^-$	20.8±10.4	37.5±34.1	24.8±11.5	21.4±15.9	33.4±28.1	27.8±8.9
$CD4^-CD8^+$	11.0±5.3	22.9±20.9	12.9±5.9	10.8±8.1	20.1±18.8	19.5±9.5
$B220^+IgM^-/IgD^-$	1.65±1.09	3.78±4.87	16.4±19.4^	10.8±11.9*	21.4±26.2	27.0±29.0
$B220^+IgM^+/IgD^+$	36.0±17.9	80.8±130	40.6±11.8	25.6±18.5	61.4±44.5	62.7±31.4°
$Mac1^+Gr1^-$	3.23±2.78	5.46±3.94	4.52±4.07	250±130***	213±168***	237±85***
$Mac1^+Gr1^+$	2.61±0.77	5.46±7.73	2.90±1.03	156±95.6***	386±142***°°°	369±101***°°°
$Mac1^+F4/80^+$	1.78±1.90	2.68±2.82	2.24±1.86	193±129***	288±199***	314±75***°°
**BM**						
**Total cells**	**64.3±19.9**	**66.1±19.1**	**68.2±22.0**	**62.9±13.9**	**74.6±19.1**	**94.1±22.2****°°°
$B220^+IgM^-/IgD^-$	3.82±2.01	2.45±1.71	6.61±5.94	0.93±1.62**	1.69±2.70	1.63±1.35*
$B220^+IgM^+/IgD^+$	5.57±2.73	6.42±4.13	8.58±7.47	0.34±0.81***	0.11±0.09***	0.16±0.17**
$Mac1^+Gr1^-$	3.43±5.75	3.58±3.03	2.63±1.27	33.3±10.1***	25.2±7.7***°	38.8±17.0***
$Mac1^+Gr1^+$	34.3±16.0	33.5±13.7	31.0±13.9	20.6±14.7*	42.1±18.4°°°	48.0±12.7**°°°
$Mac1^+F4/80^+$	17.4±13.3	15.4±14.9	16.0±15.0	28.0±16.1	34.3±19.0**	60.0±19.8***°°°
$cKit^+$	3.40±2.98	3.15±2.92	1.88±1.80	12.7±20.6	1.53±1.02	24.5±40.5
**Blood**						
**Total cells**	**9.07±2.17**	**11.8±7.0**	**19.5±22.5**	**86.9±88.2***	**303±165*****°°°	**407±196*****°°°
$CD4^+CD8^-$	2.78±0.61	3.10±1.67	3.55±2.30	8.64±13.1	11.5±21.1	8.17±3.81
$CD4^-CD8^+$	1.69±0.44	2.36±1.39	2.64±2.42	3.59±3.04	5.10±2.76*	7.10±2.59*
$B220^+IgM^-/IgD^-$	0.19±0.26	0.22±0.28	1.24±1.32^	1.34±1.28	6.89±11.6	11.1±15.9
$B220^+IgM^+/IgD^+$	3.27±1.43	4.54±3.78	9.37±14.7	3.05±1.58	9.41±5.41°	12.7±4.5°°
$Mac1^+Gr1^-$	0.67±0.21	0.92±0.46	1.48±1.06^	22.4±23.7**	113±107***°°	153±100***°°°
$Mac1^+Gr1^+$	0.74±0.37	0.83±0.38	0.72±0.27	54.7±69.2*	128±82***°	157±106***°°
$Mac1^+F4/80^+$	0.34±0.16	0.29±0.19	0.43±0.28	9.10±6.58***	34.8±31.8**°	61.6±72.8*°

Nucleated cells × 10^6, except peripheral blood cells (× 10^6/mL) from sick *MLL-AF9* AML mice and healthy GFP control mice;
n = 5-18 mice per genotype
Student's T-test with Welch's correction:
Significantly different from GFP of same genotype: ***$p \leq 0.001$; **$p \leq 0.01$; *$p \leq 0.05$
Significantly different from WT/GFP (only calculated for *Mcl-1*tg/GFP and *BCL-2*tg/GFP): ^^^$p \leq 0.001$; ^^$p \leq 0.01$; ^$p \leq 0.05$
Significantly different from WT/*MLL-AF9* (only calculated for *Mcl-1*tg/*MLL-AF9* and *BCL-2*tg/*MLL-AF9*): °°°$p \leq 0.001$; °°$p \leq 0.01$; °$p \leq 0.05$

Supplementary Table S3. Transplantation of AMLs

Genotype	1º AML mouse	AML/2º recipient	2º recipient survival (d)	2º AML mouse	AML/3º recipient	3º recipient survival (d)
**WT/*MLL-AF9***	#5	4/4	36 39 39 42			
				#10	3/3	14 14 14
	#6	5/5	13 13 13 13 13			
				#47	3/3	14 14 14
	#1501	7/7	13 13 13 13 15 17 18			
				#2347	2/2	14 16
	#1533	3/3	15 15 15			
	#1742	3/3	26 26 50			
				#2536	3/3	13 13 13
	#1745	3/3	13 14 14			
				#2557	3/3	11 12 13
	#2036	4/4	18 18 18 18			
Mcl-1*tg/*MLL-AF9	#14	5/5	28 28 33 38 38			
	#16	4/4	28 34 36 36			
				#14	3/3	14 14 14
	#17	5/5	50 50 50 50 50			
				#56	3/3	16 16 16
	#18	5/5	34 34 38 38 44			
	#1766	3/3	24 24 24			
				#2602	3/3	14 14 14
BCL-2*tg/*MLL-AF9	#19	4/4	28 28 30 33	#2		
	#22	4/4	34 34 34 34			
	#23	5/5	33 33 34 34			
				#63	3/3	25 25 33
	#24	5/5	34 34 34 34			
				#66	3/3	25 25 25
	#1563	2/2	19 19			
				#2357	3/3	15 16 16
	#2028	4/4	20 20 24 26			

Primary AMLs were injected via the tail vein into immunocompetent syngeneic secondary recipients (2 x 10^6 spleen cells / recipient for each AML) and secondary AMLs were transplanted into immunocompetent syngeneic tertiary recipients (0.5 x 10^6 bone marrow cells / recipient for AML #10, 47, 2259, 6, 14, 56, 2 and 63; 1x10^6 spleen cells / recipient for AML #2347, 2536, 2602, 2357). Table summarises efficiency of transplantation and time between transplant and ethical endpoint.

SUPPLEMENTARY REFERENCES

1. Campbell KJ, Bath ML, Turner ML, et al. Elevated Mcl-1 perturbs lymphopoiesis, promotes transformation of hematopoietic stem/progenitor cells, and enhances drug resistance. *Blood*. 2010;116(17):3197-3207.
2. Ogilvy S, Metcalf D, Print CG, Bath ML, Harris AW, Adams JM. Constitutive bcl-2 expression throughout the hematopoietic compartment affects multiple lineages and enhances progenitor cell survival. *Proc Natl Acad Sci U S A*. 1999;96(26):14943-14948.
3. Leverson JD, Phillips DC, Mitten MJ, et al. Exploiting selective BCL-2 family inhibitors to dissect cell survival dependencies and define improved strategies for cancer therapy. *Sci Transl Med*. 2015;7(279):279ra240.
4. Foucquier J, Guedj M. Analysis of drug combinations: current methodological landscape. *Pharmacol Res Perspect*. 2015;3(3):e00149.

Supplementary Figure S1 Anstee *et al*

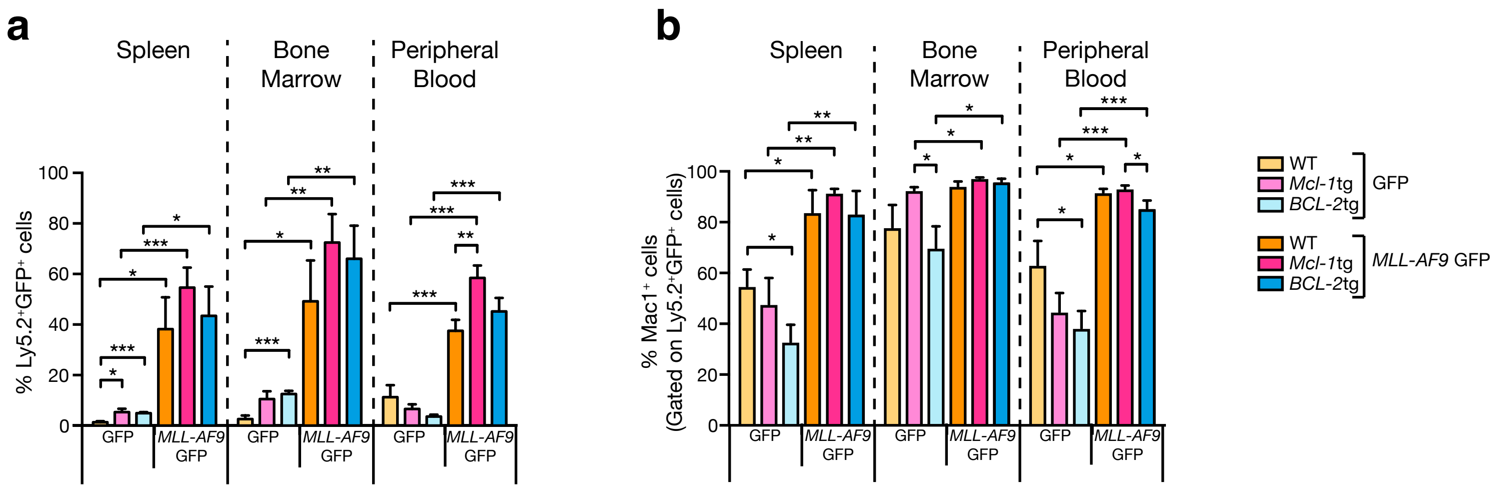

Supplementary Figure S2 Anstee *et al*

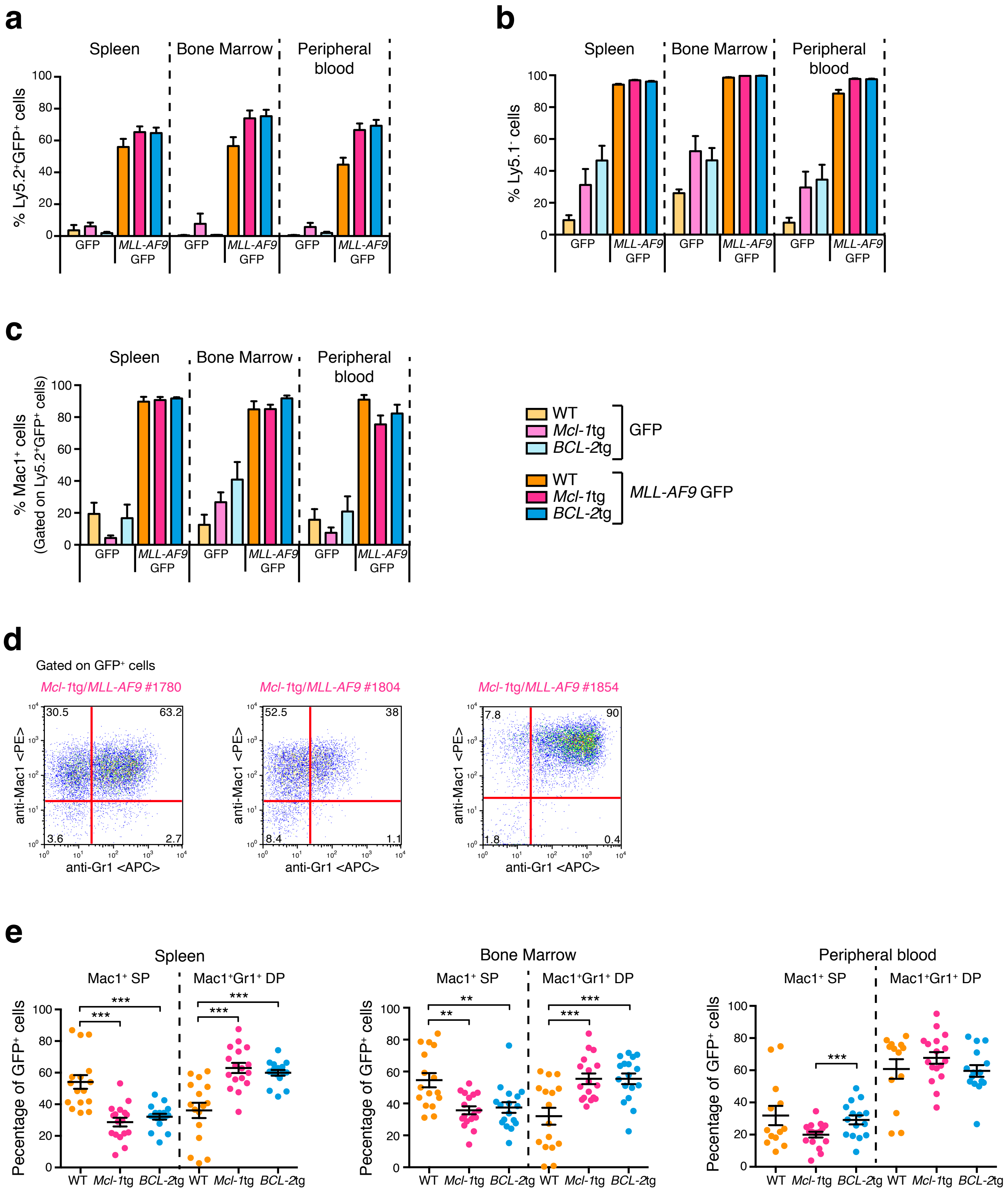

Supplementary Figure S3

Anstee *et al*

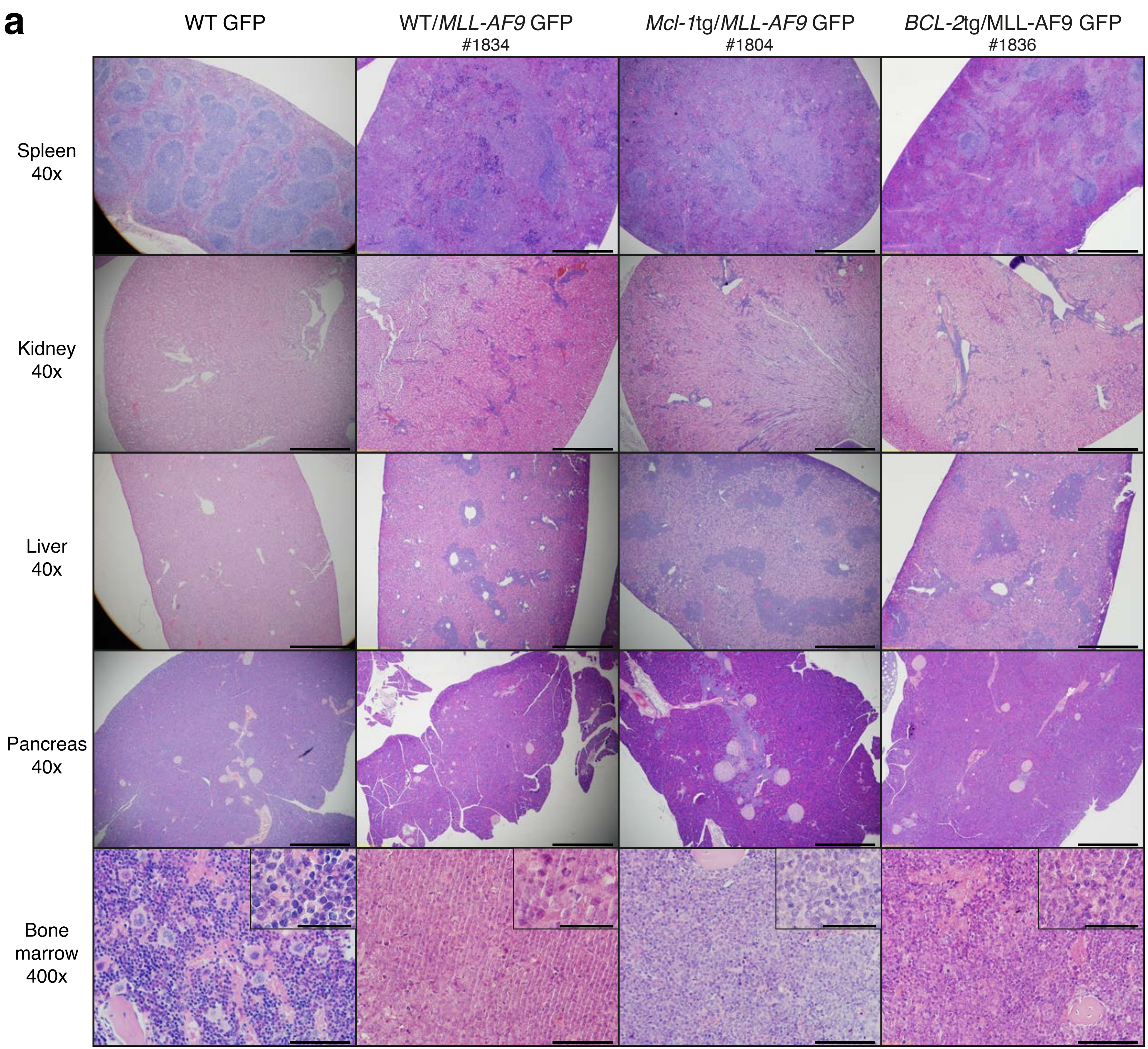

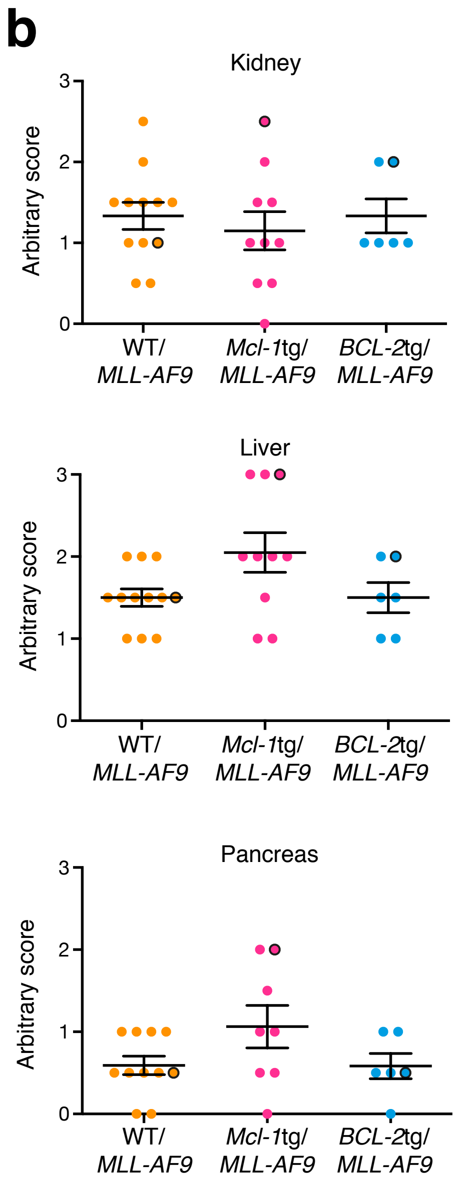

Supplementary Figure S4 Anstee *et al*

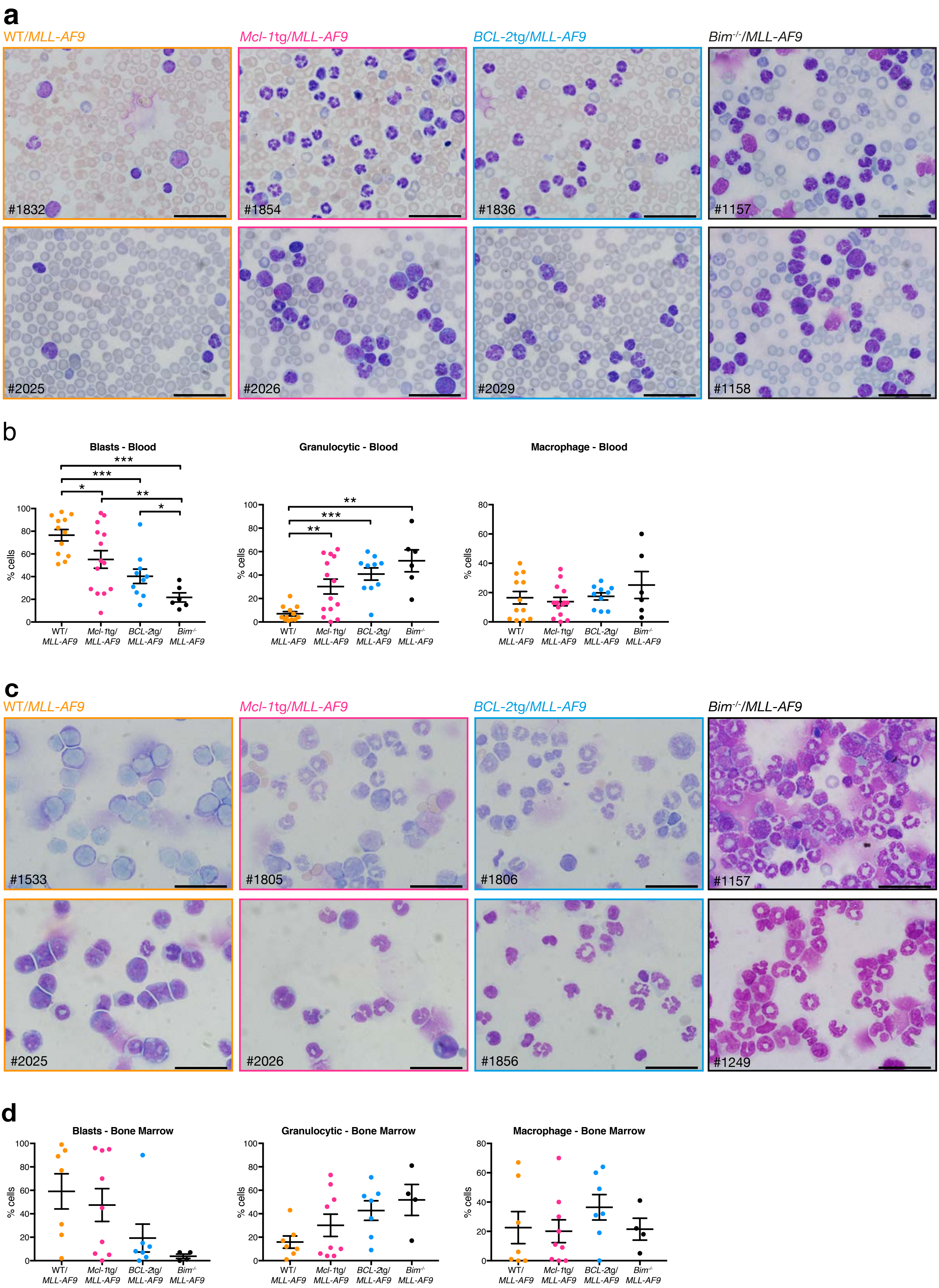

Supplementary Figure S5 Anstee *et al*

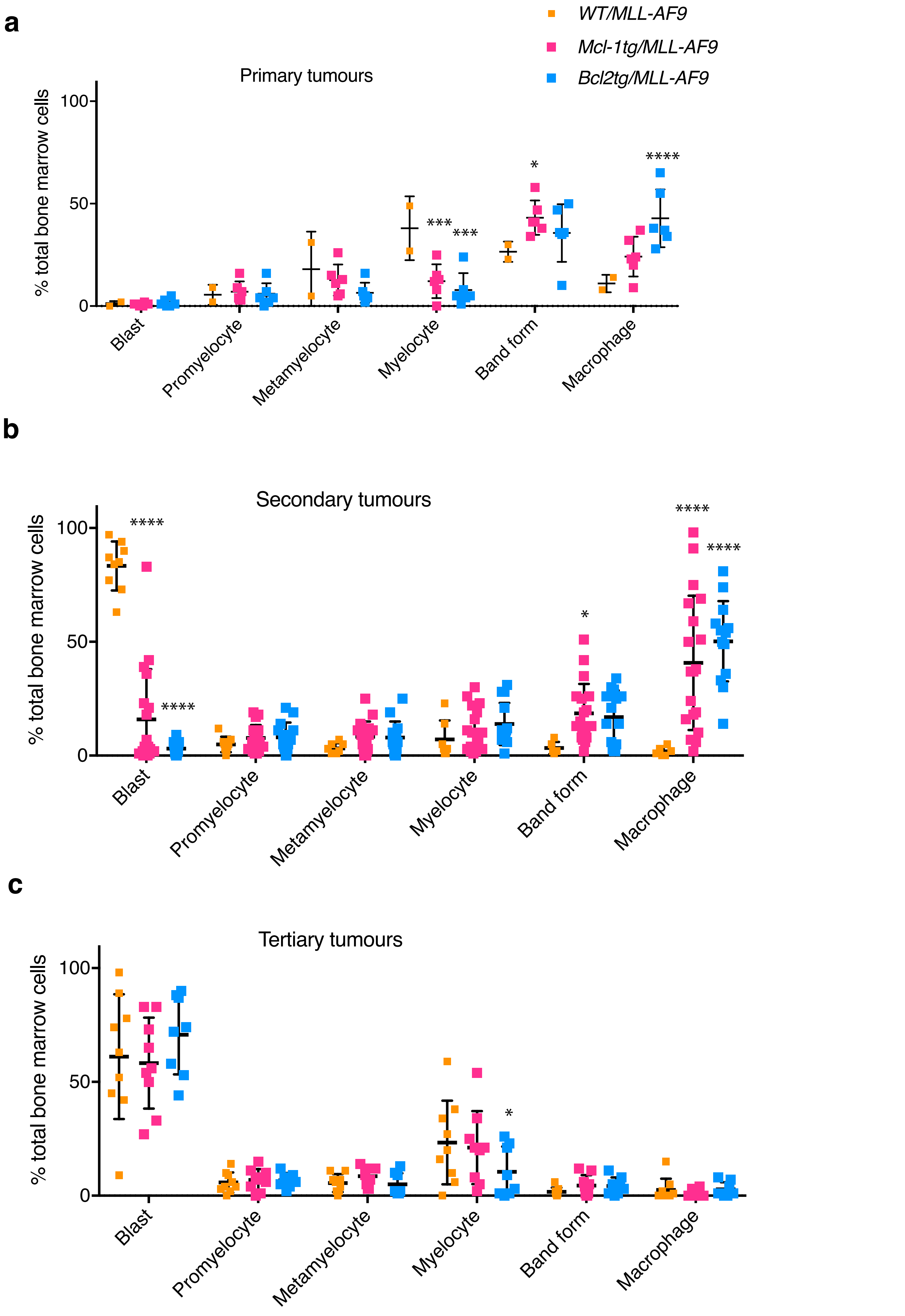

Supplementary Figure S6 Anstee *et al*

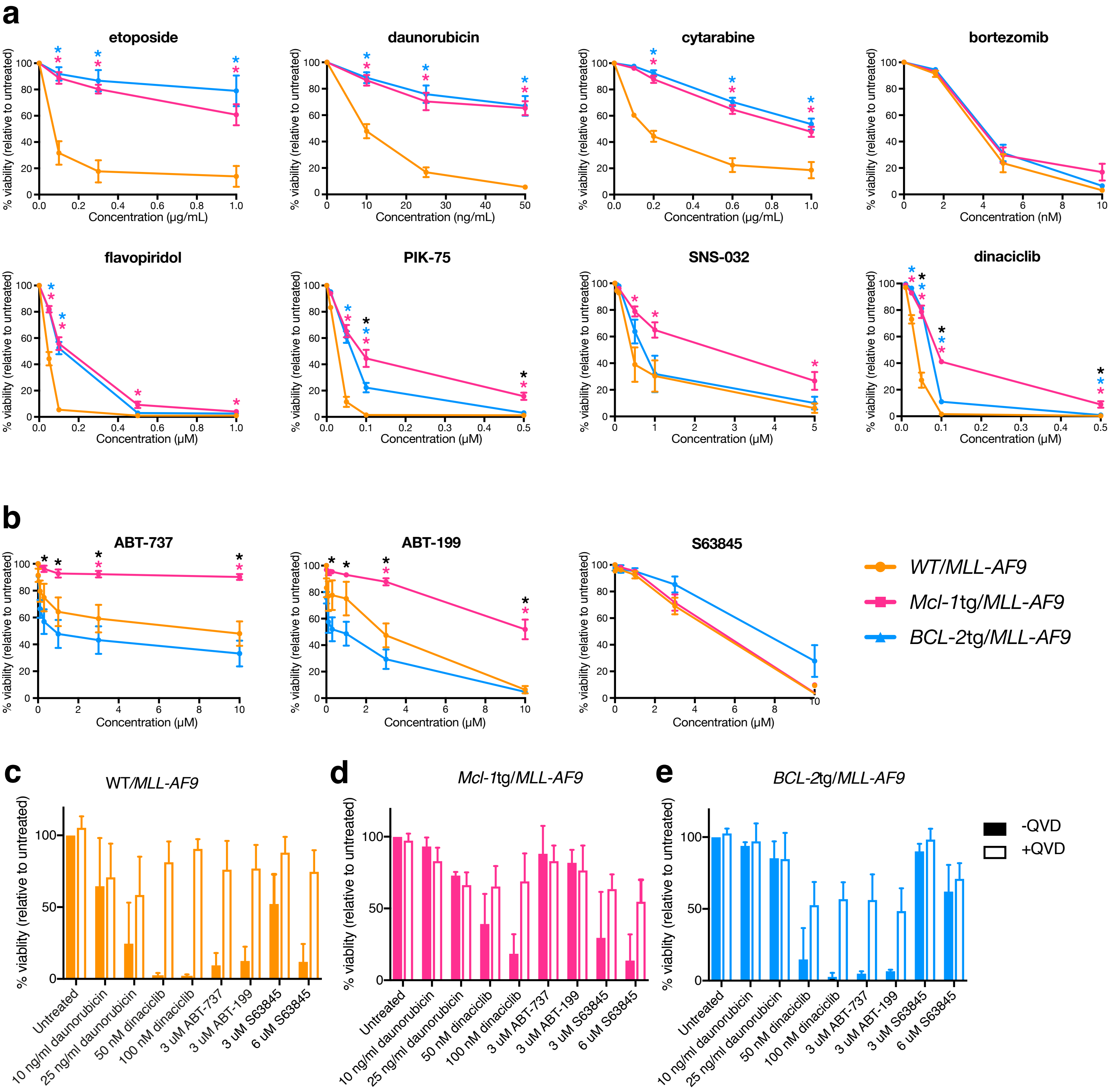

Supplementary Figure S7 Anstee *et al*

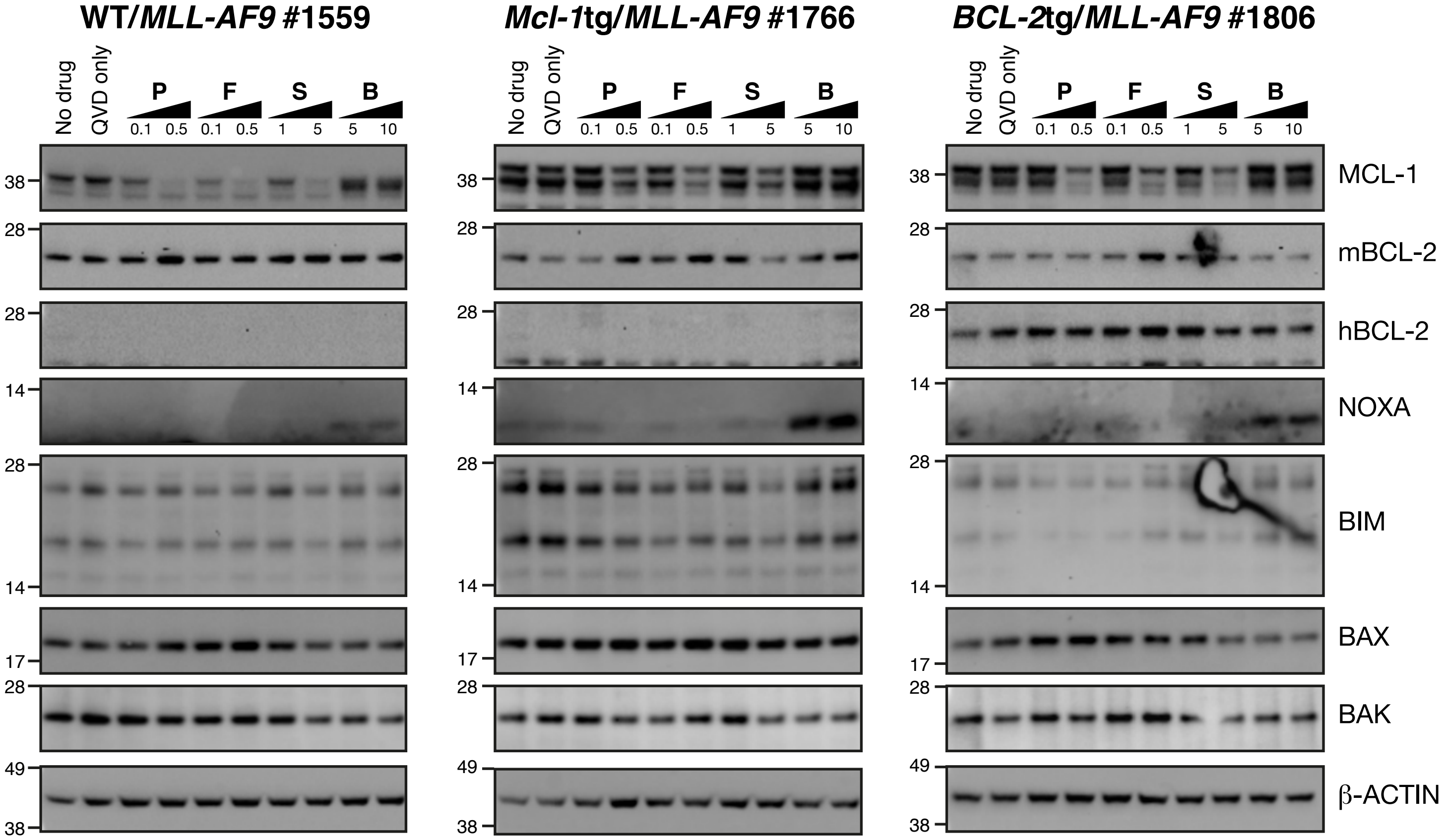

P= PIK-75 (μM) **F**= flavopiridol (μM) **S**= SNS-032 (μM) **B**= bortezomib (nM)

Supplementary Figure S8 Anstee *et al*

WT/*MLL-AF9* | *Mcl-1*tg/*MLL-AF9* | *BCL-2*tg/*MLL-AF9*

WT/*MLL-AF9*

ABT-737 (μM)	8.22	4.11	2.06	1.03	0.51	0.26	0.13	0.00
7.50	2.2	2.2	1.8	2.7	2.9	7.8	12.3	27.8
3.75	2.2	2.0	1.7	3.2	3.9	9.5	12.5	30.8
1.88	2.3	2.5	2.3	3.2	3.7	8.0	15.0	37.0
0.94	2.2	1.4	2.1	4.2	3.4	10.9	13.8	40.0
0.47	2.1	1.6	3.0	3.3	4.1	10.3	18.5	39.8
0.23	2.6	2.4	2.3	4.7	5.9	14.2	17.3	48.4
0.12	3.3	2.4	2.9	6.3	7.0	16.5	27.6	47.9
0.00	4.1	5.1	6.2	12.8	23.1	39.8	59.4	100.0

cytarabine (μM)

Bliss sum = 204.58

Mcl-1*tg/*MLL-AF9

ABT-737 (μM)	8.22	4.11	2.06	1.03	0.51	0.26	0.13	0.00
7.50	29.5	45.8	64.4	83.8	102.0	97.0	92.2	95.5
3.75	29.4	37.8	51.5	81.8	84.3	103.3	102.3	89.9
1.88	25.4	42.7	59.6	80.9	100.7	100.0	104.4	107.2
0.94	25.9	38.6	62.0	75.9	100.2	94.5	106.1	100.1
0.47	27.0	49.7	54.5	86.0	107.7	108.3	96.9	110.2
0.23	28.1	44.4	63.0	83.9	104.2	98.5	113.9	110.5
0.12	33.4	42.4	61.3	86.8	109.9	106.4	119.1	109.2
0.00	37.2	44.0	60.0	80.0	110.3	111.6	109.0	100.0

cytarabine (μM)

Bliss sum = 58.82

BCL-2*tg/*MLL-AF9

ABT-737 (μM)	8.22	4.11	2.06	1.03	0.51	0.26	0.13	0.00
7.50	1.4	1.3	1.5	2.3	2.9	5.1	3.5	9.4
3.75	1.8	1.3	1.7	1.8	2.9	4.3	7.7	13.7
1.88	1.6	1.7	2.4	2.4	2.6	4.7	8.1	14.7
0.94	2.5	1.4	2.1	2.7	3.8	8.4	8.4	14.7
0.47	2.6	1.7	3.1	3.7	4.2	7.0	11.6	21.4
0.23	3.5	3.5	4.0	4.5	6.5	9.8	16.3	29.5
0.12	4.6	3.9	5.8	8.5	11.8	16.0	20.8	31.2
0.00	35.9	44.4	57.1	80.7	85.3	93.9	92.0	100.0

cytarabine (μM)

Bliss sum = 478.04

WT/*MLL-AF9*

ABT-737 (μM)	6.00	3.00	1.50	0.75	0.38	0.19	0.09	0.00
7.50	2.7	7.9	23.8	18.2	25.3	23.4	25.8	24.0
3.75	2.2	14.5	32.0	38.0	37.2	30.9	33.6	36.3
1.88	2.5	10.2	22.2	28.2	23.6	26.9	31.3	30.1
0.94	2.4	17.6	38.8	38.7	40.2	34.8	41.2	37.8
0.47	2.7	14.1	29.0	35.0	33.2	38.6	42.1	38.4
0.23	2.6	26.1	47.8	47.2	44.3	51.8	51.0	53.3
0.12	3.0	22.1	46.8	53.1	52.1	54.5	49.8	50.2
0.00	2.5	33.5	86.1	95.4	100.5	105.6	105.5	100.0

bortezomib (nM)

Bliss sum = -87.21

Mcl-1*tg/*MLL-AF9

ABT-737 (μM)	6.00	3.00	1.50	0.75	0.38	0.19	0.09	0.00
7.50	10.0	53.2	80.6	92.3	82.9	89.8	96.8	84.1
3.75	8.2	61.7	101.1	94.7	106.2	91.0	97.4	98.0
1.88	8.4	65.5	82.2	99.7	96.4	98.9	88.5	83.9
0.94	9.3	81.0	92.6	99.6	101.3	109.6	99.8	97.4
0.47	10.1	67.0	79.7	91.7	98.6	94.3	90.9	94.1
0.23	9.8	90.4	87.0	102.9	99.7	101.2	101.7	95.5
0.12	12.2	76.1	85.1	97.6	94.7	93.9	86.6	98.0
0.00	12.9	84.1	97.4	97.4	105.2	97.0	95.0	100.0

bortezomib (nM)

Bliss sum = -49.79

BCL-2*tg/*MLL-AF9

ABT-737 (μM)	6.00	3.00	1.50	0.75	0.38	0.19	0.09	0.00
7.50	1.8	8.8	26.4	23.6	29.3	29.7	27.8	27.4
3.75	1.9	12.8	31.7	32.6	35.5	32.2	31.4	29.4
1.88	1.7	10.2	30.8	34.9	33.4	31.4	30.5	31.3
0.94	2.0	14.8	37.6	39.7	39.4	44.1	39.6	36.8
0.47	1.9	13.5	41.2	34.6	38.2	48.4	36.9	41.0
0.23	2.2	26.6	45.4	48.6	61.3	51.7	55.1	45.6
0.12	2.1	31.5	43.9	52.9	54.5	46.1	45.1	59.6
0.00	5.2	72.0	83.0	72.3	95.8	89.2	101.3	100.0

bortezomib (nM)

Bliss sum = 81.07

WT/*MLL-AF9*

ABT-737 (μM)	500.00	250.00	125.00	62.50	31.25	15.63	7.81	0.00
7.50	2.0	1.6	1.6	2.1	5.7	14.1	21.8	25.2
3.75	1.7	1.5	1.4	2.3	7.3	16.3	23.8	30.9
1.88	2.1	1.7	1.6	2.0	5.1	15.7	26.4	30.5
0.94	2.2	1.7	1.3	2.2	8.1	22.6	29.0	36.0
0.47	2.1	1.5	1.3	2.1	7.0	21.8	31.0	39.2
0.23	2.2	1.5	1.6	2.3	12.2	30.8	47.4	52.3
0.12	2.1	1.5	1.6	2.9	13.1	28.8	46.3	47.5
0.00	2.5	1.6	2.0	5.0	28.4	62.9	87.0	100.0

flavopiridol (nM)

Bliss sum = 40.68

Mcl-1*tg/*MLL-AF9

ABT-737 (μM)	500.00	250.00	125.00	62.50	31.25	15.63	7.81	0.00
7.50	12.0	25.6	41.9	61.2	100.9	104.4	100.4	100.3
3.75	12.5	22.4	33.1	56.0	92.9	100.2	104.8	95.6
1.88	13.8	27.8	44.8	59.9	87.4	98.1	106.3	97.1
0.94	11.8	23.0	36.9	68.4	87.3	109.4	103.9	112.4
0.47	13.5	23.7	50.3	61.6	87.9	110.6	112.5	107.4
0.23	12.8	25.4	34.3	63.0	99.3	118.5	102.8	123.2
0.12	13.8	24.6	41.2	67.1	94.0	104.9	102.0	106.2
0.00	16.3	28.5	40.8	72.6	84.9	118.5	110.3	100.0

flavopiridol (nM)

Bliss sum = 7.44

BCL-2*tg/*MLL-AF9

ABT-737 (μM)	500.00	250.00	125.00	62.50	31.25	15.63	7.81	0.00
7.50	1.7	1.2	1.3	2.1	8.8	12.6	24.3	24.3
3.75	1.7	1.2	1.4	2.2	9.6	21.8	26.9	37.9
1.88	1.9	1.3	1.2	1.9	9.6	21.0	29.3	36.4
0.94	1.9	1.5	1.3	2.8	12.6	26.9	36.7	37.6
0.47	2.1	1.5	1.5	3.0	15.3	31.9	43.6	47.6
0.23	2.2	1.7	2.3	5.7	23.7	50.3	54.9	62.9
0.12	2.5	1.7	3.8	3.7	26.9	63.0	78.1	87.0
0.00	2.6	4.1	12.4	43.4	81.9	86.3	105.5	100.0

flavopiridol (nM)

Bliss sum = 536.74

WT/*MLL-AF9*

ABT-737 (μM)	100.00	50.00	25.00	12.50	6.25	3.13	1.56	0.00
7.50	1.5	1.6	4.9	13.6	19.8	16.6	17.5	15.1
3.75	1.8	1.6	11.1	23.8	27.4	28.5	27.1	26.4
1.88	1.7	1.4	4.1	12.4	20.4	20.4	19.9	19.6
0.94	1.8	1.6	11.3	23.9	37.3	29.8	36.4	34.1
0.47	2.0	1.5	3.9	15.0	25.4	26.4	27.8	25.6
0.23	2.0	1.9	16.0	30.7	47.3	49.0	47.4	44.0
0.12	1.7	1.9	4.5	22.0	31.6	38.4	34.3	41.3
0.00	1.9	2.7	31.7	80.7	88.1	89.5	96.5	100.0

dinaciclib (nM)

Bliss sum = 36.39

Mcl-1*tg/*MLL-AF9

ABT-737 (μM)	100.00	50.00	25.00	12.50	6.25	3.13	1.56	0.00
7.50	7.7	29.6	72.2	89.4	83.6	95.5	93.0	84.4
3.75	9.4	49.4	86.3	104.3	90.2	102.1	101.0	99.1
1.88	7.8	31.2	69.4	87.4	84.2	91.3	88.3	82.8
0.94	10.1	52.3	87.3	93.4	96.9	91.3	103.1	92.3
0.47	7.8	33.6	63.2	92.8	95.7	91.9	92.1	95.3
0.23	9.7	51.3	78.7	95.2	100.5	109.4	91.8	105.0
0.12	8.5	31.5	60.6	85.3	98.9	97.0	96.0	101.1
0.00	12.2	51.1	79.0	97.3	100.4	105.2	101.6	100.0

dinaciclib (nM)

Bliss sum = -28.41

BCL-2*tg/*MLL-AF9

ABT-737 (μM)	100.00	50.00	25.00	12.50	6.25	3.13	1.56	0.00
7.50	1.8	1.5	2.3	5.8	6.7	7.0	6.9	6.4
3.75	1.9	1.2	4.1	9.1	9.8	11.3	11.7	10.1
1.88	1.8	1.4	2.6	6.4	7.4	8.2	7.9	7.5
0.94	1.9	1.4	6.1	11.0	13.5	15.9	16.2	13.6
0.47	1.8	1.3	3.3	9.8	11.4	13.6	14.2	13.0
0.23	1.6	1.6	8.2	16.1	24.3	23.7	24.9	21.5
0.12	1.7	1.9	5.6	18.9	26.2	25.9	25.5	25.0
0.00	2.0	26.2	78.9	90.8	92.3	93.4	98.0	100.0

dinaciclib (nM)

Bliss sum = 41.36

WT/*MLL-AF9*

ABT-737 (μM)	500.00	250.00	125.00	62.50	31.25	15.63	7.81	0.00
7.50	1.7	1.3	1.0	1.4	1.7	2.3	8.8	31.0
3.75	1.7	1.2	1.2	1.1	1.5	3.5	11.0	29.1
1.88	1.5	1.5	1.1	1.3	1.8	4.6	14.6	30.6
0.94	1.7	1.6	1.2	1.4	1.6	5.4	13.8	36.6
0.47	1.8	1.3	1.3	1.4	2.1	7.3	15.9	39.4
0.23	1.9	1.3	1.4	1.2	2.5	8.3	20.5	42.7
0.12	2.2	1.6	1.3	1.3	2.0	6.1	20.1	56.8
0.00	2.3	1.3	1.3	1.5	3.6	20.9	53.8	100.0

PIK-75 (nM)

Bliss sum = 42.68

Mcl-1*tg/*MLL-AF9

ABT-737 (μM)	500.00	250.00	125.00	62.50	31.25	15.63	7.81	0.00
7.50	7.5	17.1	23.9	28.1	44.8	68.2	80.9	87.3
3.75	7.3	14.8	20.5	23.2	42.0	72.9	95.8	102.2
1.88	7.7	17.7	21.0	26.1	46.4	82.9	96.5	93.8
0.94	7.3	14.1	18.1	28.0	50.1	75.9	92.2	104.8
0.47	8.2	16.9	21.9	29.6	51.5	78.0	97.4	95.4
0.23	7.9	15.3	21.1	27.2	55.3	85.2	96.8	105.7
0.12	9.4	17.0	23.5	27.4	50.5	79.5	105.9	103.8
0.00	9.9	17.4	23.5	31.9	56.1	84.4	93.7	100.0

PIK-75 (nM)

Bliss sum = 88.74

BCL-2*tg/*MLL-AF9

ABT-737 (μM)	500.00	250.00	125.00	62.50	31.25	15.63	7.81	0.00
7.50	1.2	1.0	0.9	1.1	1.5	2.2	3.7	9.9
3.75	0.9	0.9	0.9	1.0	1.4	2.2	5.2	11.0
1.88	0.9	0.9	0.9	1.0	1.4	2.5	5.6	10.7
0.94	1.1	1.1	0.8	1.0	2.1	3.6	7.3	15.1
0.47	1.0	1.0	1.1	1.2	1.6	5.0	7.9	19.2
0.23	1.4	1.1	0.9	1.4	2.6	6.7	11.8	20.6
0.12	1.4	1.1	1.1	1.6	3.1	6.6	18.6	34.6
0.00	2.0	1.9	4.2	21.4	53.6	69.1	96.2	100.0

PIK-75 (nM)

Bliss sum = 201.40

Supplementary Figure S9 Anstee *et al*

WT/*MLL-AF9*

cytarabine (µM) → / S63845 (µM) ↓	8.22	4.11	2.06	1.03	0.51	0.26	0.13	0.00
3.00	2.8	2.5	3.4	10.3	16.2	41.9	52.8	63.1
1.50	2.2	3.7	11.5	18.1	43.8	55.9	77.5	98.5
0.75	5.2	4.2	11.1	25.1	42.7	71.5	83.7	103.6
0.38	2.6	5.0	10.8	24.5	48.9	75.6	86.9	102.2
0.19	4.9	4.7	11.2	25.9	50.4	70.7	96.4	98.3
0.09	3.3	5.9	10.1	27.0	46.2	68.8	92.8	100.3
0.05	5.5	5.4	16.0	27.1	52.5	75.8	96.6	103.8
0.00	3.5	7.1	11.9	25.0	50.2	75.9	72.9	100.0

Bliss sum = 3.17

bortezomib (nM) → / S63845 (µM) ↓	6.00	3.00	1.50	0.75	0.38	0.19	0.09	0.00
3.00	3.2	27.5	36.9	40.4	36.6	40.8	41.1	35.4
1.50	7.2	77.6	81.4	79.2	87.1	89.8	78.4	83.3
0.75	6.1	76.8	91.1	85.6	85.6	93.7	84.7	93.2
0.38	7.2	82.2	91.7	96.8	94.8	100.7	99.8	99.6
0.19	6.6	83.1	85.9	94.0	103.9	101.7	86.9	96.6
0.09	9.3	86.1	102.2	99.3	106.7	106.5	100.1	105.1
0.05	6.6	81.5	94.4	102.6	96.3	94.9	100.5	95.4
0.00	6.1	90.6	103.0	100.1	99.5	101.3	113.0	100.0

Bliss sum = -109.64

flavopiridol (nM) → / S63845 (µM) ↓	500.00	250.00	125.00	62.50	31.25	15.63	7.81	0.00
3.00	2.3	1.7	1.7	3.2	16.1	30.2	41.3	43.6
1.50	2.6	1.9	1.7	9.9	34.9	55.6	64.9	67.1
0.75	3.0	2.0	2.2	11.1	44.2	69.7	70.8	73.5
0.38	2.9	2.2	2.5	15.7	54.7	78.3	86.9	94.8
0.19	3.0	2.3	2.7	12.3	51.6	74.4	90.2	90.9
0.09	3.4	2.2	2.5	12.0	66.7	84.3	86.5	89.5
0.05	3.3	2.3	3.1	17.0	59.5	87.6	89.2	91.4
0.00	2.9	2.0	2.5	14.1	56.6	80.5	92.3	100.0

Bliss sum = -27.20

dinaciclib (nM) → / S63845 (µM) ↓	100.00	50.00	25.00	12.50	6.25	3.13	1.56	0.00
3.00	2.0	2.6	7.7	24.0	40.6	42.8	42.7	45.4
1.50	2.2	4.0	30.5	78.0	87.1	95.1	92.2	82.8
0.75	2.2	5.5	23.3	58.2	81.1	76.0	78.1	81.5
0.38	2.2	5.3	42.3	89.2	99.8	100.9	95.1	91.1
0.19	2.3	4.7	19.4	68.5	79.4	85.8	88.6	83.9
0.09	2.5	5.4	51.2	91.0	95.6	100.0	91.3	94.0
0.05	2.4	7.1	21.5	72.2	91.0	95.6	87.0	86.0
0.00	2.2	3.5	49.2	85.3	94.9	101.1	91.3	100.0

Bliss sum = 181.53

PIK-75 (nM) → / S63845 (µM) ↓	500.00	250.00	125.00	62.50	31.25	15.63	7.81	0.00
3.00	2.6	1.5	1.4	1.5	2.6	9.2	34.6	54.8
1.50	2.8	1.8	1.5	1.7	4.3	32.9	67.5	84.0
0.75	3.0	1.9	1.6	1.7	6.5	46.2	75.2	89.1
0.38	3.1	1.8	1.5	1.9	12.0	46.6	71.1	95.0
0.19	2.9	2.2	1.6	1.8	12.3	55.0	80.9	96.6
0.09	3.0	2.1	1.7	1.8	15.5	57.9	86.1	96.5
0.05	3.2	2.1	1.7	1.9	10.5	40.1	84.6	100.7
0.00	2.9	1.8	1.7	1.8	11.2	45.8	76.6	100.0

Bliss sum = 2.51

*Mcl-1*tg/*MLL-AF9*

cytarabine (µM) → / S63845 (µM) ↓	8.22	4.11	2.06	1.03	0.51	0.26	0.13	0.00
3.00	3.7	9.4	19.3	29.8	39.1	53.6	57.3	58.5
1.50	7.0	20.9	32.0	49.4	78.5	86.8	77.3	94.2
0.75	14.2	24.4	48.0	63.8	82.8	95.5	89.4	96.6
0.38	11.6	23.4	43.6	69.4	74.7	96.6	93.6	96.6
0.19	16.0	24.8	52.4	75.0	88.3	99.7	108.1	99.7
0.09	11.2	24.4	37.1	55.9	94.7	83.0	100.8	102.9
0.05	14.9	23.9	39.6	64.1	94.5	84.6	98.5	105.0
0.00	13.2	24.7	47.9	76.9	84.7	92.0	91.3	100.0

Bliss sum = 140.49

bortezomib (nM) → / S63845 (µM) ↓	6.00	3.00	1.50	0.75	0.38	0.19	0.09	0.00
3.00	4.9	27.2	46.6	47.8	53.0	48.4	59.8	56.1
1.50	9.5	73.6	83.9	90.8	91.8	96.9	95.1	81.2
0.75	9.6	74.5	98.8	97.1	88.2	93.7	92.5	96.8
0.38	15.9	87.9	100.4	99.7	101.3	99.9	98.4	101.9
0.19	13.4	85.4	96.2	98.8	97.3	100.5	91.3	99.0
0.09	21.3	85.7	103.6	103.6	99.1	100.4	104.1	102.3
0.05	15.6	84.2	99.0	94.0	99.6	102.4	101.1	98.6
0.00	15.7	85.7	96.3	101.9	100.9	101.6	102.7	100.0

Bliss sum = -50.08

flavopiridol (nM) → / S63845 (µM) ↓	500.00	250.00	125.00	62.50	31.25	15.63	7.81	0.00
3.00	3.3	3.8	6.0	16.2	27.3	51.3	39.7	45.4
1.50	5.6	10.7	23.5	46.9	60.0	84.4	90.8	95.4
0.75	8.4	23.6	39.3	59.2	78.9	102.0	91.9	95.1
0.38	14.7	31.1	47.5	67.6	85.8	92.3	97.9	97.7
0.19	12.5	35.1	51.8	64.1	84.4	102.4	91.6	97.3
0.09	13.5	43.4	48.5	68.8	91.6	94.5	107.6	95.1
0.05	20.3	38.0	48.2	71.4	87.7	98.5	105.3	95.7
0.00	18.8	34.8	45.9	63.6	73.4	99.7	91.2	100.0

Bliss sum = 157.06

dinaciclib (nM) → / S63845 (µM) ↓	100.00	50.00	25.00	12.50	6.25	3.13	1.56	0.00
3.00	2.9	7.5	33.3	53.7	59.7	58.3	53.8	51.1
1.50	9.0	39.6	67.6	92.3	96.4	94.3	93.2	85.2
0.75	11.3	35.5	74.6	87.0	95.6	94.8	93.2	89.8
0.38	14.7	43.1	78.3	99.0	95.4	103.7	100.0	98.0
0.19	15.8	36.0	76.4	94.9	92.5	97.1	97.8	97.4
0.09	16.7	48.1	87.8	95.3	96.4	97.7	100.2	94.8
0.05	16.2	40.4	74.0	95.9	99.0	95.1	98.4	98.3
0.00	15.2	41.5	83.2	101.5	100.4	97.0	102.5	100.0

Bliss sum = 24.06

PIK-75 (nM) → / S63845 (µM) ↓	500.00	250.00	125.00	62.50	31.25	15.63	7.81	0.00
3.00	3.0	2.3	2.5	4.5	14.2	31.3	52.6	53.9
1.50	4.0	7.4	10.9	18.6	35.6	77.1	89.9	92.9
0.75	9.5	16.0	19.3	27.8	56.5	88.1	105.4	101.0
0.38	14.5	16.7	24.4	33.6	60.6	92.9	102.5	97.9
0.19	15.8	23.8	26.3	34.8	60.8	95.2	101.6	100.7
0.09	16.4	24.6	25.3	33.4	61.9	96.3	100.3	100.0
0.05	17.9	27.7	28.2	32.9	57.4	84.7	99.0	102.7
0.00	16.6	23.4	26.8	37.6	57.9	81.1	110.1	100.0

Bliss sum = 143.22

*BCL-2*tg/*MLL-AF9*

cytarabine (µM) → / S63845 (µM) ↓	8.22	4.11	2.06	1.03	0.51	0.26	0.13	0.00
3.00	10.0	8.3	14.3	26.1	39.4	41.7	45.3	69.7
1.50	6.9	8.6	18.6	36.7	50.3	76.8	84.7	78.0
0.75	8.8	8.6	18.6	44.2	60.0	80.0	89.0	93.5
0.38	7.7	14.7	17.8	44.7	63.4	79.3	92.5	107.2
0.19	14.5	11.0	21.7	48.8	57.9	86.2	90.0	97.8
0.09	8.3	16.0	32.1	41.7	74.5	100.3	84.8	93.6
0.05	16.0	11.5	28.1	47.7	60.2	94.9	107.0	93.6
0.00	7.6	14.2	29.1	44.1	77.4	82.9	98.9	100.0

Bliss sum = 190.07

bortezomib (nM) → / S63845 (µM) ↓	6.00	3.00	1.50	0.75	0.38	0.19	0.09	0.00
3.00	3.4	34.0	54.0	59.6	53.0	56.9	63.1	64.2
1.50	10.1	81.5	90.4	87.7	101.1	99.1	98.9	99.1
0.75	9.1	84.6	100.3	88.1	107.9	97.3	99.9	96.9
0.38	15.4	92.9	105.9	112.2	104.7	105.5	112.8	108.7
0.19	11.1	87.7	105.6	101.2	107.1	110.8	107.1	106.1
0.09	17.7	102.9	114.4	117.0	113.5	107.8	104.2	117.1
0.05	11.9	94.4	97.6	111.3	102.4	108.2	99.0	96.4
0.00	13.8	97.4	109.8	106.5	106.9	106.2	112.3	100.0

Bliss sum = 43.44

flavopiridol (nM) → / S63845 (µM) ↓	500.00	250.00	125.00	62.50	31.25	15.63	7.81	0.00
3.00	2.6	2.3	4.3	14.2	39.7	49.0	58.4	64.6
1.50	2.9	3.0	7.0	24.8	55.9	67.2	74.5	76.2
0.75	3.3	3.7	8.6	25.8	64.6	82.5	88.4	96.1
0.38	3.6	3.6	11.9	30.7	70.0	88.3	89.1	95.0
0.19	3.6	3.9	13.8	28.5	64.3	86.6	84.3	94.4
0.09	3.5	4.7	11.7	30.4	57.1	85.8	91.8	99.3
0.05	4.0	4.0	16.3	41.0	72.2	82.8	101.3	96.4
0.00	3.2	4.2	15.2	31.9	55.7	87.0	88.1	100.0

Bliss sum = 70.91

dinaciclib (nM) → / S63845 (µM) ↓	100.00	50.00	25.00	12.50	6.25	3.13	1.56	0.00
3.00	2.1	3.5	25.2	48.6	54.8	54.8	52.9	63.4
1.50	2.4	17.1	51.6	82.5	81.1	92.7	84.3	77.1
0.75	2.6	9.1	54.2	72.6	87.4	92.3	82.9	80.6
0.38	2.9	15.4	64.8	87.5	88.6	91.5	99.3	87.8
0.19	2.6	11.2	56.2	78.1	88.9	90.8	83.1	91.7
0.09	2.9	27.6	72.0	92.8	88.6	92.4	96.0	86.7
0.05	2.6	16.3	52.4	83.9	88.2	91.6	90.1	89.5
0.00	2.8	20.6	68.4	81.4	90.4	90.2	97.1	100.0

Bliss sum = 87.72

PIK-75 (nM) → / S63845 (µM) ↓	500.00	250.00	125.00	62.50	31.25	15.63	7.81	0.00
3.00	2.9	2.3	2.6	5.7	18.2	38.6	51.0	55.9
1.50	3.2	2.2	3.3	8.9	27.1	60.5	73.3	83.2
0.75	3.3	2.6	3.7	8.2	32.8	77.8	100.9	93.0
0.38	3.8	3.1	5.1	15.3	47.0	77.1	101.7	92.2
0.19	3.8	3.7	5.1	15.1	42.2	86.2	104.0	113.1
0.09	3.9	3.2	5.0	14.4	50.5	88.6	96.7	106.4
0.05	4.3	3.4	4.7	13.3	40.9	81.7	106.4	119.0
0.00	3.8	3.1	4.4	15.0	41.4	76.6	106.9	100.0

Bliss sum = 2.60
